# Symbiont Identity Impacts Prokaryotic Microbiome Dynamics During Heat Stress in a Model System for Corals

**DOI:** 10.1093/icb/icag086

**Published:** 2026-06-26

**Authors:** Victoria M Glynn, Evan C Lawrence, Phillip A Cleves, Rowan D H Barrett

**Affiliations:** Department of Biology and Redpath Museum, McGill University, Montréal, Québec H3A 1B1, Canada; Naos Marine Laboratories, Smithsonian Tropical Research Institute (STRI), Ancón, Panamá, Republic of Panama; Department of Embryology, Carnegie Institution for Science, Baltimore,MD 21218, USA; Department of Embryology, Carnegie Institution for Science, Baltimore,MD 21218, USA; Department of Biology and Redpath Museum, McGill University, Montréal, Québec H3A 1B1, Canada

## Abstract

The coral microbiome is highly complex, and interactions between microbiome members have been proposed as an important component of coral thermotolerance. However, establishing causal links among specific microbiome members remains difficult in corals because these communities are diverse and difficult to manipulate experimentally. We used *Aiptasia*, an emerging coral model system, to test how algal symbiont identity influences the structure and dynamics of the prokaryotic microbiome during heat stress. We generated clonal *Aiptasia* lines hosting two well-defined strains of photosynthetic algae in the family Symbiodiniaceae. We exposed these animals to an acute thermal stress assay while tracking prokaryotic dynamics with 16S sequencing. Among heat-stressed animals, algal strain was the strongest driver of prokaryotic community composition. We also identified line-associated indicator taxa that may be linked to differences in bleaching resistance. Finally, the more thermally sensitive host-algal association showed greater intersample dissimilarity in prokaryotic community structure under heat stress, suggesting that sustained microbiome variability may characterize more stress-sensitive cnidarian holobionts. These results suggest algal symbionts may shape bleaching responses not only through effects on host physiology, but also through their influence on prokaryotic microbiome dynamics.

## Introduction

Climate change is increasing the frequency and severity of heat waves, placing coral reefs that support over 25% of all marine life at great risk (Hoegh-Guldberg et al. 2000; Ayre and Hughes 2004; Donovan et al. 2021; Wyatt et al. 2023). The foundation of reef ecosystems is the symbiotic relationship between the coral host and photosynthetic algae in the family Symbiodiniaceae. It is predicted that this single partner is responsible for approximately 90% of the host’s energy requirements in most coral species (Grottoli et al. 2006; Jones et al. 2008). This obligate symbiosis breaks down during heat stress, and the host expels its algal symbionts in a process known as coral bleaching (Grottoli et al. 2006; Graham et al. 2007; Jones et al. 2008; Pratchett et al. 2008; LaJeunesse et al. 2018; Stuart-Smith et al. 2018). Algal symbionts may play a dual role in also supporting the host’s own ability to withstand thermal stress. For example, *Durusdinium* spp. algae are predominantly associated with corals reported to best survive thermal stress. Furthermore, *Durusdinium* spp. algae are linked to increasing the host’s thermal thresholds by as much as 1.5°C (Glynn et al. 2001; Berkelmans and van Oppen 2006; Silverstein, Cunning and Baker 2017; Claar et al. 2020). Algal symbionts that evolved under heat stress have also been shown to increase the host’s thermotolerance (Buerger et al. 2020; Chan et al. 2023). Corals can thus provide a useful case study for how host–microbiome interactions are central to host homeostasis, and in turn impact ecosystem-level processes.

The coral microbiome extends beyond Symbiodiniaceae to also include bacteria, protists, viruses, archaea, endoliths, and fungi (Peixoto et al. 2021). Bacteria are perhaps one of the most well-studied microbiome members besides Symbiodiniaceae, with certain members posited to be beneficial to the host during bleaching, while others are putative agents of coral disease (Kline and Vollmer 2011; Gardner et al. 2019; Doering et al. 2023; Rosales et al. 2023). Variation in prokaryotic communities has also been shown to correlate with divergent bleaching trajectories, and in turn inoculating corals with bacterial cultures with specific genetic and/or phenotypic characteristics can potentially reduce bleaching and pathogen presence (Ziegler et al. 2017; Rosado et al. 2019; Santoro et al. 2021; Cárdenas et al. 2022; Xu et al. 2023; Delgadillo-Ordoñez et al. 2024). The coral microbiome is a dynamic entity, with algae and prokaryotes strongly impacting host health, and likely one another (see Peixoto et al. 2021). However, the precise functional links between microbiome members have been challenging to ascertain, in part due to the inherent complexity of these communities.

*Aiptasia* is a tractable anthozoan model for coral symbiosis because aposymbiotic animals can be experimentally reinfected with defined Symbiodiniaceae strains, allowing host genetic background and algal symbiont identity to be manipulated more readily than in most corals. Aposymbiotic anemones can be reinfected with a variety of Symbiodiniaceae species including those from stony corals (Weis et al. 2008; Hambleton et al. 2014; Baumgarten et al. 2015; Grawunder et al. 2015), allowing for the creation of clonal lines differing in their hosted algal symbiont. Changing these algal symbionts can lead to dramatic shifts in bleaching tolerance, suggesting a role for the microbiome in controlling the animal stress response (Xiang et al. 2025). Additionally, *Aiptasia* and stony corals are both anthozoan cnidarians, but *Aiptasia* lack a calcareous skeleton, a feature that facilitates tissue extraction, microscopy, and fluorescent analyses (Lehnert et al. 2012; Baumgarten et al. 2015). In addition to their algal symbionts, *Aiptasia*, like corals, have a prokaryotic microbiome, but the role of these members in overall holobiont functioning is poorly understood, particularly within the context of bleaching. Previous research has shown that aposymbiotic and symbiotic *Aiptasia* associate with different prokaryotic members, suggesting that algal symbionts may be impacting the community composition of other microbiome members (Röthig et al. 2016). Additionally, symbiotic state can impact *Aiptasia* bacterial community dynamics under thermal stress (Aguirre et al. 2023; Sydnor et al. 2023), different symbionts can impact metabolomic and proteomic responses under thermal stress (Lust et al. 2025), and symbiotic *Aiptasia* of different clonal backgrounds can have different prokaryotic communities (Ahmed et al. 2019). Moreover, animals of the same clonal background (CC7) hosting different algal strains (SSA01 versus SSB01) also associate with different prokaryotic communities after being exposed to long-term elevated temperatures (Ahmed et al. 2019). How algal symbiont identity influences both *Aiptasia*’s overall prokaryotic community dynamics and bleaching severity at the onset of heat stress has not been directly tested. Doing so may reveal prokaryotic taxa associated with bleaching resilience at a resolution currently unavailable in corals, improving our understanding of the microbiome’s role in coral thermotolerance.

In this study, we explored how algal thermotolerance impacts *Aiptasia*’s prokaryotic community dynamics. To do so, we subjected *Aiptasia* clonal lines hosting different defined strains of photosynthetic algae to an acute thermal stress assay known to result in bleaching (Cleves et al. 2020) and monitored the prokaryotic community over time with 16S sequencing. We predicted that different host–algae associations would result in divergent prokaryotic communities, as the algal symbiont’s thermal characteristics would shape overall microbiome structure and function. Additionally, we expected that particular prokaryotic taxa would be differentially associated with different time points in our assay, thus emerging as particular indicator species for bleaching severity. By leveraging *Aiptasia* as an emerging model system for coral biology, we can experimentally manipulate the cnidarian microbiome to gain insights into how algal symbionts impact prokaryotic dynamics during heat stress.

## Materials and methods

### Organisms and culture conditions

We generated all animals from a clonal population of CC7 *Aiptasia* animals, which naturally form symbioses with *Symbiodinium linucheae* (Sunagawa et al. 2009; Bieri et al. 2016). We first rendered these animals aposymbiotic, following the previously established combined cold-shock and diuron protocol (see Lehnert et al. 2012; Xiang et al. 2013). We then exposed the aposymbiotic CC7 animals to clonal, axenic strains of either Symbiodiniaceae strain SSA01 (*S. linucheae*, ITS2 Clade A4) or SSB01 (*Breviolum minutum*, ITS2 Clade B1); these clonal strains have been described previously (Xiang et al. 2013; Bieri et al. 2016). To create and confirm our algal strains were axenic, we used growth on rich media (casein hydrolysate and marine broth), following Xiang et al. 2013. The resulting animal lines will be referred to herein as CC7-SSA01 and CC7-SSB01. We grew *Aiptasia* under standard laboratory conditions (see below) for at least two years prior to our experiment to ensure that algal populations were in a steady state. We periodically sequenced cps23S (chloroplast rDNA), 18S (nuclear rDNA), and/or ITS2 (nuclear rDNA), to ensure animals remained in symbiosis with their clonal algal strains (Xiang et al. 2013). We maintained all animals at 27°C in artificial seawater (ASW), which we prepared by mixing Coral Pro Salt (Red Sea) with deionized water (dH_2_O), for a final salinity of 33.5 ppt. We kept animals under a 12 h:12 h light: dark cycle at an irradiance of ∼25 μmol photons m^−2^ s^−1^ (Phillips Alto II 25 W white fluorescent bulbs) and fed them twice weekly with freshly hatched *Artemia nauplii*, with a subsequent water change at the end of feeding.

### Heat stress experimental set-up

**Fig. 1:**
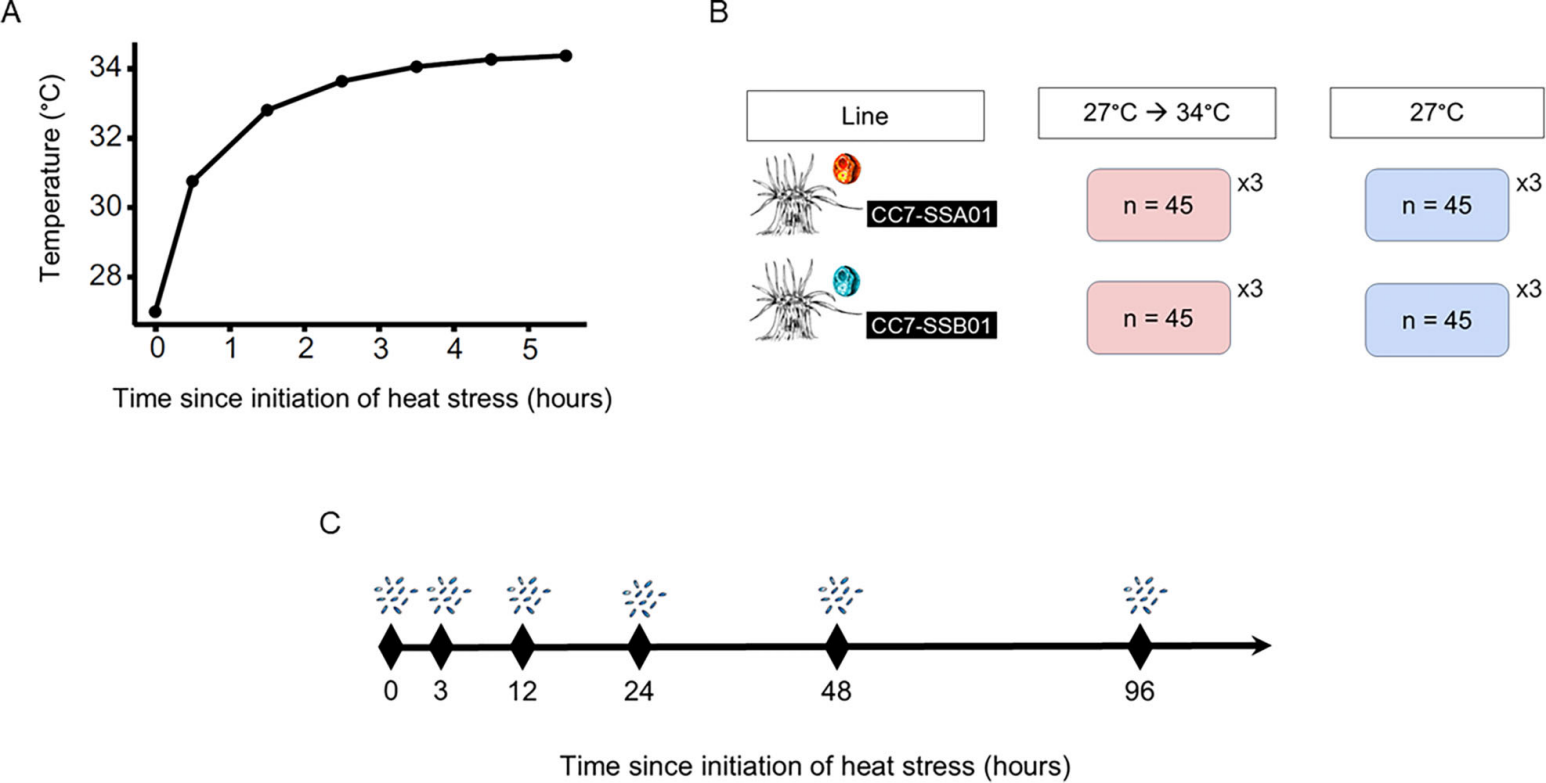
Overview of the experimental design for the *Aiptasia* prokaryotic heat stress experiment. The two *Aiptasia* lines used were CC7-SSB01 and CC7-SSA01. (A) Temperature ramp for the heat stress treatment, following Cleves et al. (2020). Animals were moved from their original incubators at 27°C to a 34°C incubator. This temperature increase occurred over the course of 5 hours. (B) Tank set-up for the experiment. To prevent overcrowding the animals, three separate tanks with 45 individuals each were subjected to our temperature ramp up. Three additional tanks remained at 27°C to serve as our controls. (C) Overview of sampling time points over the course of our heat stress assay. Note that given our temperature ramp-up, at *t* = 3 h animals had not reached the final target temperature of 34°C.

To explore prokaryotic shifts during thermal stress, we subjected CC7-SSA01 and CC7-SSB01 animals to the heat stress assay described in Cleves et al. (2020). Briefly, we placed 45 animals from each line in a polycarbonate tank with 1 L of ASW. For each line, we had a total of six tanks: three for our heat stress assay, and three to serve as our control. These animals were collected from our larger stocks and allowed to acclimate in our experimental incubators for 2 weeks at 27°C prior to the start of the experiment. At the end of the two-week acclimation period, we began our heat stress assay. Approximately 3 h into their light cycle, 3 animals were pooled per line to serve as our *t* = 0 h samples. Immediately after, three tanks per line were moved from their original incubator at 27°C to one at 34°C, with an approximate 5-h ramp up to reach the final experimental temperature. We also prepared a tank with 1 L of ASW and a HOBO Pendant Temperature Logger to record water temperatures throughout the experiment at 5-minute intervals. One tank with a HOBO logger was kept with the control animals at 27°C, and another tank with a HOBO logger was moved from 27°C to 34°C alongside our heat stress treatment animals to track the increase in temperature. To account for tank effects, one animal from each of the three replicate heat stress tanks, for each line, was pooled at *t* = 0, 3, 12, 24, 48, and 96 h and placed in 1.5 mL of DNA/RNA Shield (Zymo Research) for downstream microbiome characterization. Given space limitations, heat-stress and control treatments were conducted in two sequential experimental batches rather than simultaneously. Both batches used the same source cultures and husbandry conditions, identical acclimation duration and light cycle, the same sampling schedule and preservation protocol, and the same downstream DNA extraction, sequencing parameters, and analysis workflows. Water temperature was continuously logged during each batch to verify the intended thermal profiles. We note that treatment and batch are therefore confounded, and we interpret temperature-associated patterns conservatively in light of this design constraint. Fig. 1 provides an overview of our experimental design.

### DNA extraction and 16S amplification

To characterize prokaryotic dynamics during heat stress, we performed amplicon sequencing of the *Aiptasia* prokaryotic community. From each sample, we extracted DNA using a modified phenol-chloroform protocol we optimized for cnidarians. Briefly, we first lysed whole animals in their stored DNA/RNA Shield (Zymo Research) using a combination of Lysing Matrix A and 1/4-inch ceramic spheres (MP Biomedicals). This was followed by enzymatic digestion using both proteinase K and RNAse A, and a standard phenol: chloroform: isoamyl phase separation. The resulting DNA was shipped to the McGill Genome Centre (Montréal, Québec) for amplicon sequencing of the 16S rRNA marker-gene using the Earth Microbiome Project’s updated 515F (5′-GTGYCAGCMGCCGCGGTAA-3′; Parada et al. 2016) and 806R (5′-GGACTACNVGGGTWTCTAAT-3′; Apprill et al. 2015) primer pair (Ul-Hasan et al. 2019; https://earthmicrobiome.org/protocols-and-standards/16s/). As samples were submitted in two separate batches, corresponding to heat stress and control samples (see *Heat stress experimental set-up*), this resulted in the McGill Genome Centre using slightly different amplification protocols, albeit using the same primers and downstream Illumina sequencing protocols. For the heat stress samples, both 16S amplification and barcoding were performed in a single PCR step using KAPA HiFi 2X Ready Mix (x30 cycles). For the control samples, while the abovementioned one-step PCR was initially tested, this did not yield optimal results. Therefore, our control samples followed a two-step PCR approach. The first PCR used a Phusion HF/GC Buffer and Phusion DNA polymerase for 16S amplification (x30 cycles), followed by a separate PCR barcoding step with the 2X Kapa HiFi HotStart ready mix (x12 cycles). The full details regarding both samples PCR conditions can be found in Table S1 and S2. While the same sequencing workflow was used for all samples, these differences in PCR conditions could impact downstream bioinformatic analyses. However, given our analyses focused on heat stress samples alone, and we r-log transformed our data prior to our beta diversity analyses, run-level differences were accounted for and therefore do not significantly impact our overall conclusions regarding prokaryotic dynamics for our heat stressed animals. All amplicons were sequenced on an Illumina MiSeq v2, with a paired-end read length of 250 bp, aiming for approximately 100,000 reads per amplicon.

### Characterizing prokaryotic dynamics under thermal stress

To determine prokaryotic taxonomy and community dynamics from our 16S reads, we used the R package “DADA2” version 1.36.0 (Callahan et al. 2016; http://github.com/benjjneb/dada2) to call amplicon sequence variants (ASVs). Here, we used cutadapt in DADA2 to remove primer sequences (Martin 2011). We also assigned taxonomy using the SILVA 16S database release v138.2 (Quast et al. 2013; Callahan 2024), specifically formatted for the classification of ASVs derived from DADA2 (https://zenodo.org/records/14169026). Using the R package “phyloseq” (McMurdie and Holmes 2013), we removed all ASVs assigned to chloroplasts, mitochondria, eukaryotes, or unassigned at the kingdom level. We used the package “decontam” (Davis et al. 2018) to remove potential contaminants within our ASV data, by implementing a statistical classification procedure on the basis of prevalence within our negative controls. To do so, we identified contaminant ASVs, with the prevalence-based method at threshold 0.5. We additionally removed singletons and performed an r-log transformation within the “DESeq2” package (Love et al. 2014) prior to beta diversity analyses. All analyses were performed using R version 4.5.2 (R Core Team 2025).

To determine community-level shifts during thermal stress, we performed a Principal Coordinate Analysis (PCoA) via the “ordinate” function in “phyloseq” (McMurdie and Holmes 2013), using Bray-Curtis dissimilarity to calculate distances (Bray and Curtis 1957). To help identify the impact of each explanatory variable in driving patterns within ordination space, we ran a permutational multivariate analysis of variance (PERMANOVA) with 999 permutations using the “adonis2” command from the package “vegan” (Dixon 2003); post hoc pairwise comparisons were performed with the “pairwise.adonis2” function and implemented Benjamini and Hochberg *P*-value corrections due to multiple comparisons (Martinez Abizu 2020). To test the statistical significance of patterns of homogeneity of variance, we ran “betadisper” with 999 permutations from the package “vegan,” using Bray–Curtis distances (Dixon 2003). We then ran an ANOVA on the “betadisper” group dispersion results to determine if the variance of each group’s distance from the centroid was statistically significant. Post hoc comparisons were performed with Tukey’s Honest Significant Differences test, which corrects for multiple testing at a 95% confidence level.

To detect differentially abundant microorganisms across *Aiptasia* lines and time points, we performed an indicator species analysis (Dufrêne and Legendre 1997) using the package “indicspecies” (Cáceres and Legendre 2009) and used a modified function from Setiawan et al. 2022 that groups our ASV table into higher order taxonomy; for our analyses, we grouped our ASV table down to the genus level. As a result, the input table for “indicspecies” has collapsed the ASV counts into counts across different genera represented in our data. Using this genera table, we used the “multipatt” command with 999 permutations and implemented the Benjamini and Hochberg *P*-value correction for multiple comparisons. As we were most interested in differentially associated taxa during heat stress, we focused only on our heat stress samples in these analyses. It is important to note that “multipatt” follows the conceptual underpinnings of Cáceres and Legendre (2009), where a species is denoted as an indicator species on the basis of both specificity and fidelity, balancing both its relative abundance when comparing our two groups, as well as how frequently it appears within replicates of a given group. As such, these identified species are not necessarily those with the highest relative abundances, but rather those who are the most robust indicators of a given group’s microbiome. As we did not find strong support for prokaryotic microbiome changes as a function of time (see Results), we compared lines with all time points pooled together.

To determine differences in *Aiptasia* prokaryotic communities across samples under heat stress, we calculated sample similarity networks using “igraph” (Csárdi et al. 2026; https://doi.org/10.5281/zenodo.20471759). We used a fixed Bray–Curtis threshold (0.1) to generate networks with adequate connectivity for summary metrics while preserving the major sample-structuring patterns observed in ordinations (see Results). For each line’s network, we calculated its density, transitivity, average path length, and maximal clique size, which are all indicators of community connectivity. To determine the likelihood of each specific line’s network (e.g., CC7-SSA01 versus CC7-SSB01) and its associated metrics, we created 1000 random graphs using an Erdös–Rényi model and determined the proportion of graphs with a shorter path length than the original graph (Erdös and Rényi 2011). Here, randomly generated graphs have the same number of vertices and density as the original network whereas the location where the edges are drawn is allowed to vary. For network-to-network metric comparisons, we used the random network’s metric values to calculate our null distribution’s means and standard deviations. This was then used in a two-tailed *z*-score test to determine if calculated metrics were statistically different between networks.

## Results

### *Aiptasia* line strongly structures prokaryotic community dynamics under heat stress

**Fig. 2:**
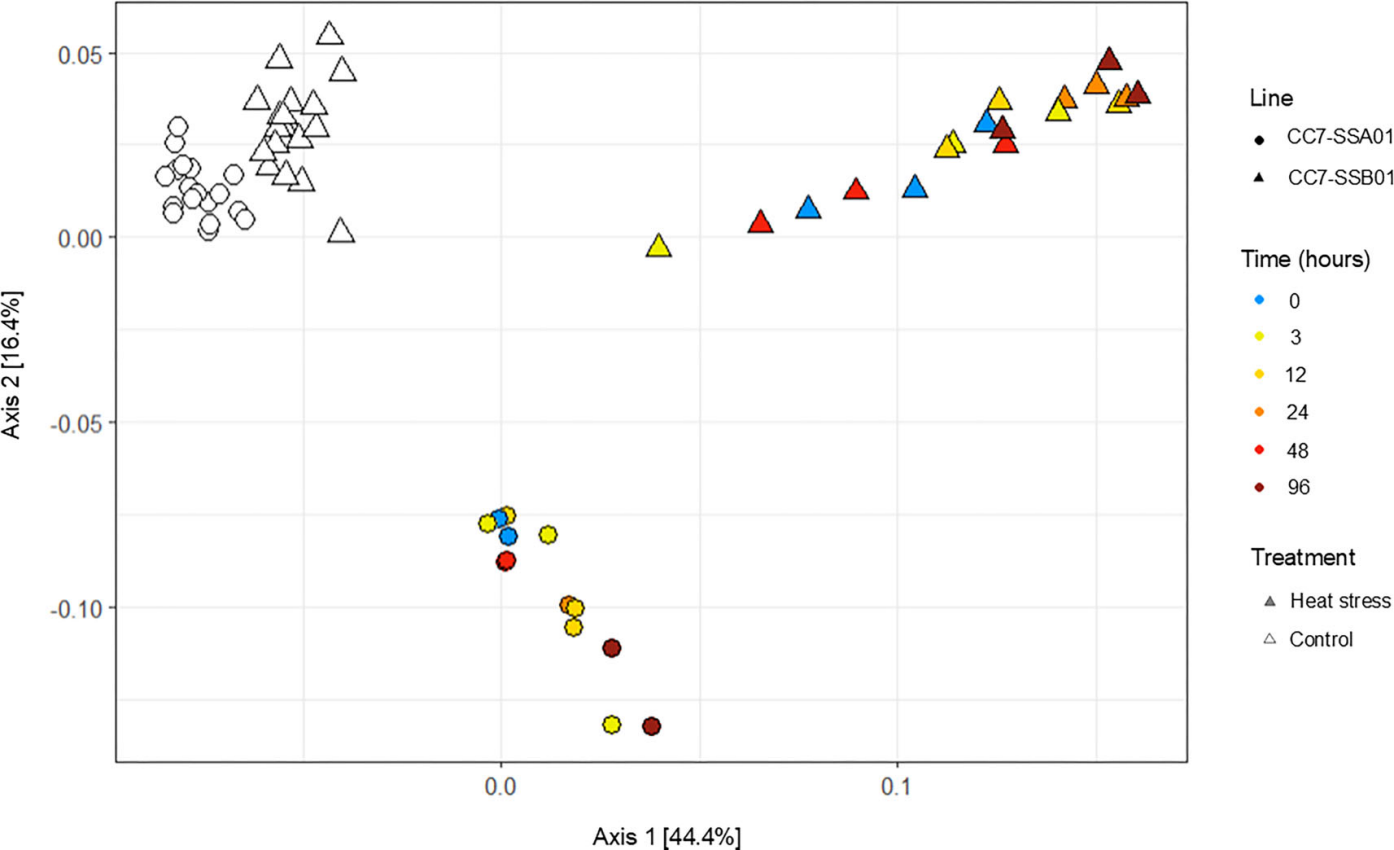
Principal coordinate analysis (PCoA) for *Aiptasia*’s prokaryotic communities over the course of heat stress, based on Bray–Curtis dissimilarity in the relative abundances of ASVs. Colors indicate different time points, while shapes represent one of the two *Aiptasia* lines used. Control samples are showed as hollow points, while heat-stressed samples are shown as solid colored points.

PCoA plots reveal that over the duration of the assay, CC7-SSA01 and CC7-SSB01 animals associated with distinct prokaryotic communities, with the duration of heat stress producing additional within-cluster structure among samples. Furthermore, the control samples appear to have a prokaryotic microbiome that is distinct from the treatment animals. (Fig. 2). All animals were drawn from the same larger line-specific stock cultures and acclimated for two weeks under identical conditions, making the observed line-associated differences notable (see Methods). PC1 discriminates between our control and treatment animals, with some fine-scale structure between the two lines, while PC2 predominantly reflects changes that occur over time under heat stress. The PERMANOVA revealed that treatment, time, strain, and the (1) interaction of treatment and time and (2) the interaction of treatment and strain significantly shaped the prokaryotic microbiome (*P* < 0.05; see Table S3). Comparison of effect sizes on the basis of the pseudo-*F*-statistic revealed that treatment (*P* = 0.001, pseudo-*F*-statistic_43,66_ = 82.4287, *R*^2^ = 0.36344) was the strongest driver of prokaryotic community comparison across all statistically significant factors, followed by line (*P* = 0.001, pseudo-*F*-statistic_43,66_ = 37.5002, *R*^2^ = 0.16534). Given the strong treatment effect and our experiment’s goals of ascertaining prokaryotic dynamics under thermal stress, we focused on heat stress animals for subsequent analyses.

When considering heat stressed animals alone, line (*P* = 0.001, pseudo-*F*-statistic_19,30_ = 52.1688, *R*^2^ = 0.56290) and time (*P* = 0.006, pseudo-*F*-statistic_19,30_ = 2.9972, *R*^2^ = 0.16170) separately emerge as significantly driving patterns in community similarity, with line emerging as the strongest driver of prokaryotic community composition on the basis of the pseudo-*F*-statistic; interestingly, the interaction between time and line emerged as nonsignificant (Table S4). Pairwise post hoc comparisons revealed that when comparing across time points for each line separately, no two time points resulted in statistically distinct community compositions (Tables S5 and  S6). This suggests a modest overall temporal signal that does not resolve into consistent pairwise separations among specific time points after correction. Furthermore, we did not find strong evidence that CC7-SSA01 animals or CC7-SSB01 animals have differences in community variance or distance from centroid across time when subjected to heat stress (Tables S7 and S8). Together, this suggests that while some fine-scale restructuring may be occurring over the course of the assay, line is the main driver of prokaryotic dynamics. This was unexpected as over the course of our experiment, the two lines demonstrated significant changes in algal cell densities as a function of time, which is a direct measure of bleaching; here, CC7-SSA01 animals overall experienced less bleaching overall over the 96-h experiment (see Fig. S1, Tables S9 and S10).

### Line-associated indicator taxa emerge over the duration of thermal stress

In focusing on heat stress animals, indicator species analyses revealed that different taxa were differentially associated with each line during our assay. We ran these analyses with all time points grouped within each line, as our previous analyses did not provide strong support that individual time points had distinct prokaryotic communities. Out of 196 identified taxa, 102 were associated with CC7-SSA01 animals and 94 were associated with CC7-SSB01 animals (Table S11). While Alphaproteobacteria and Gammaproteobacteria taxa were both seen to differentially associate with our lines, alongside different Phycisphaerales and Chlamydiales species, some notable distinctions emerge. For instance, for the CC7-SSA01 line, *Thalassococcus* sp., *Maricaulis* sp., *Pseudomonas* sp., Myxococcales, and *Halobacteriovorax* taxa emerge as being line-associated indicator taxa. Conversely, for CC7-SSB01 animals, Sphingobacteriales, *Oceanospirillum* sp., *Marinobacter* sp., and *Vibrio* sp. taxa were differentially associated with this line. Many of the taxa distilled by our indicator species analyses have been previously identified as microbiome members of stony corals and *Aiptasia*; some of these taxa have been proposed to impact the susceptibility of their hosts to environmental stressors (see Discussion). It is important to note that when calculating the mean relative abundance of these various taxa within each line, this value did not exceed 0.03 (see Table S11). Yet given how indicator species are calculated, this is relevant and underscores their categorization as such. Indicator taxa are meant to be strong indicators of differences between our two *Aiptasia* lines, and therefore are not expected to be most highly abundant taxa but rather those able to discriminate between them.

### Prokaryotic communities of CC7-SSB01 animals are more variable between samples

**Fig. 3:**
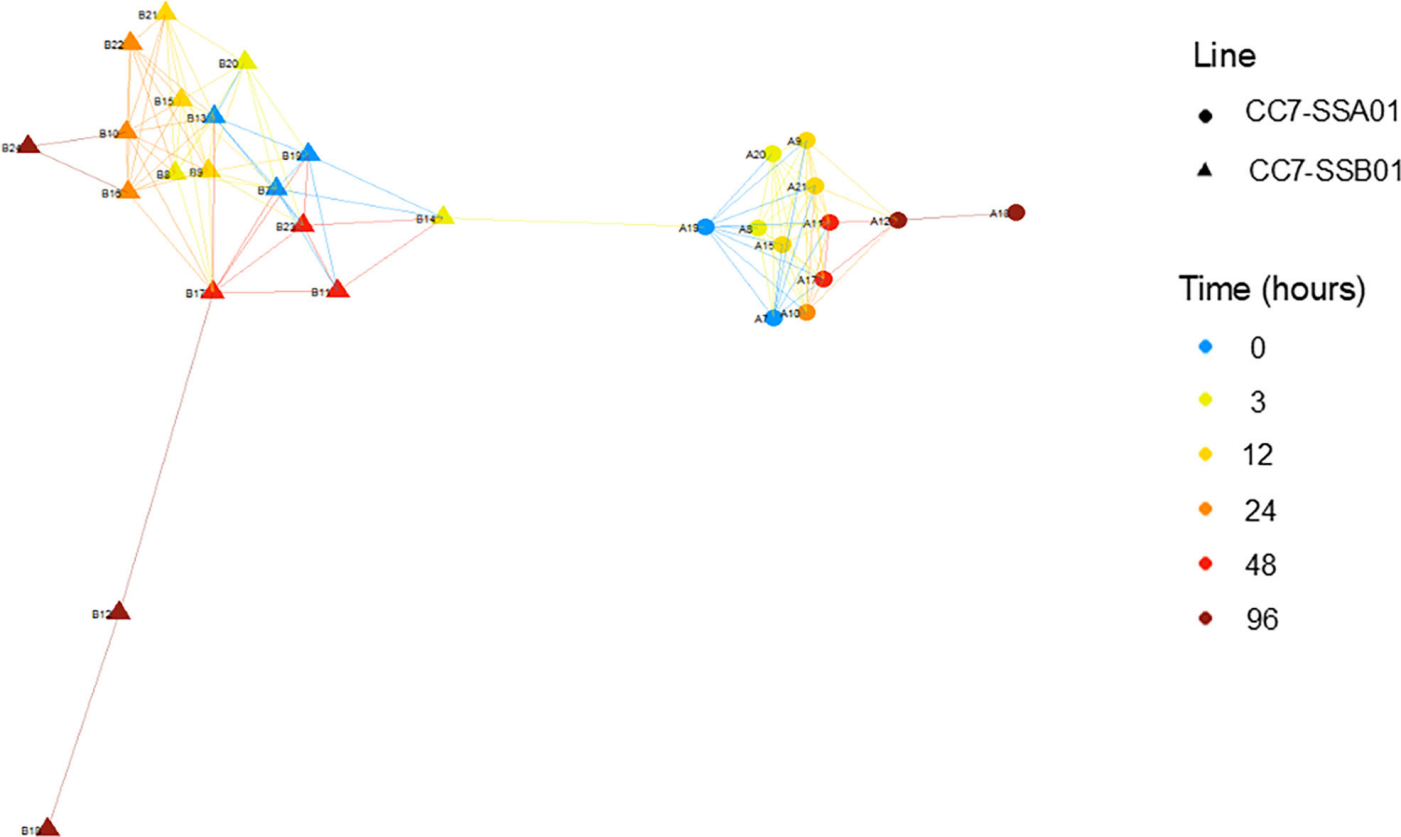
Sample similarity networks for *Aiptasia* under heat stress. Plots implement Bray–Curtis dissimilarity to define links between the nodes, here corresponding to individual samples, with a distance threshold of 0.1. Colors indicate different time points, while shapes represent the two *Aiptasia* lines used.

Network analyses underscore how under heat stress, our lines displayed inherent differences in the connectivity of their prokaryotic communities. The prokaryotic community of CC7-SSA01 animals appeared to be more connected than for CC7-SSB01 animals, as demonstrated by the longer path length between samples for CC7-SSB01 animals (Fig. 3). This observation was further supported when calculating network metrics for the CC7-SSA01 and CC7-SSB01 plots, where the average path length was longer between samples for CC7-SSB01 animals as compared to CC7-SSA01 animals (*P* < 0.05; Tables S12-S13). For our other metrics of interest, network density, transitivity, and maximum clique size, these were not statistically distinct between the CC7-SSA01 and CC7-SSB01 networks (all three metrics *P* > 0.05; Table S12). Counter to our expectations, when formally testing the significance of observed trends, only the CC7-SSB01 animals had path lengths that were statistically distinct from that of the randomly generated networks (*P* < 0.05). For all other metrics, each line had significantly different characteristics as compared to their corresponding random networks, apart from network density for each line respectively (Table S13). While each line’s realized network was distinct from the null expectation, a notable distinction emerges in the case of path length, which can be interpreted as CC7-SSB01 samples being more dissimilar over the course of the assay as compared to CC7-SSA01.

## Discussion

By directly comparing clonal *Aiptasia* lines that vary in their associated algal symbiont, our data indicate that algal symbiont identity may intrinsically shape prokaryotic community dynamics. There are differences in the specific prokaryotic taxa associated with CC7-SSA01 and CC7-SSB01 animals under thermal stress, with those associated with CC7-SSB01 animals having been previously identified as differentially abundant in heat-stressed cnidarians and are linked to coral diseases like stony coral tissue loss disease (cf. Bourne et al. 2008; Ahmed et al. 2019; Huntley et al. 2022). Thus, our data suggest that the presence of these taxa is characteristic of an inherently more environmentally sensitive cnidarian holobiont. Future work should explore these taxa from the perspective of health indicators, and their potential correlations to overall bleaching and/or disease severity in cnidarians. Furthermore, our network analyses suggest that the prokaryotic communities of CC7-SSB01 animals were more dissimilar to one another across time points than for CC7-SSA01 animals. These network metrics posit that greater intersample variability may be indicating weaker host control on microbial dynamics (Zaneveld et al. 2017). Together, our findings suggest that algal symbionts may be impacting host thermotolerance beyond strictly metabolic interactions (Cantin et al. 2009; Rädecker et al. 2018; Sproles et al. 2019; Allen-Waller and Barott 2023; Lust et al. 2025) by also potentially shaping prokaryotic microbiome dynamics under thermal stress.

### Thermal stress and hosted algal symbiont jointly impact *Aiptasia* prokaryotic dynamics

Heat stress and hosted algal strain structured *Aiptasia*’s prokaryotic communities over the course of our assay. Previous work in corals has shown how both species identity and abiotic conditions shape the microbiome (Ziegler et al. 2019; Haydon et al. 2021; Glynn et al. 2023; Glynn et al. 2025). In explicitly controlling for host genetics by having clonal *Aiptasia* lines, we found that treatment, followed by line, were the main drivers of community composition. Additionally, in considering each line separately under thermal stress, our post hoc comparisons did not reveal significant differences in prokaryotic community composition between pairs of time points for either CC7-SSA01 or CC7-SSB01 animals, suggesting that these differences may have been capturing more global strain-specific changes. This indicates that, in addition to genetic background, algal symbionts may also shape the associated prokaryotic microbiome of their hosts (see Aguirre et al. 2023).

Recent work in *Aiptasia* suggests that symbiont strain identity may influence the host’s proteomic and metabolic responses under thermal stress and that antibacterial treatments impact animal reproduction, highlighting the importance of algal–prokaryotic interactions in cnidarian fitness (Lust et al. 2025; McCauley et al. 2025). Our study serves as a confluence point to prior work, by tracking at a higher resolution both prokaryotic and bleaching dynamics over time, while controlling for host genetic background and hosted algal strain. Algal–prokaryotic interactions are widely regarded as an important component of coral fitness (Matthews et al. 2020; Mohamed et al. 2023), and *Aiptasia* is an ideal system to manipulate symbiotic associations and ascertain how they shape overall holobiont functioning and bleaching pathways under stress.

A limitation of our design is that while all animals originate from the same source cultures, the control and heat-stress assays were run sequentially because of incubator space constraints, such that batch and treatment are not fully separable. We minimized potential batch-related variation by standardizing acclimation, light cycle, sampling schedule, and all downstream processing, and by continuously logging temperatures to confirm the intended profiles. Importantly, our main conclusions focus on (1) consistent differences between the two *Aiptasia* lines and (2) microbiome restructuring observed across the heat-stress time series within each line, which are less plausibly explained by generic batch effects alone. Nevertheless, we interpret treatment-associated differences conservatively and view these results as strong motivation for future experiments in which control and heat stress are run in parallel.

### Line-associated prokaryotes provide candidates for functional testing

Indicator analyses highlighted line-specific taxa under heat stress that provide candidates for health indicators and probiotic testing (Table S9). These were not the most abundant members of either line’s microbiomes, not exceeding a mean relative abundance of 0.03 within each line, respectively (see Table S9), thus potentially revealing nuanced changes within these animals’ symbiotic relationships and health status. CC7-SSA01 was differentially associated with taxa reported to have putative antimicrobial or predatory potential (e.g., *Pseudomonas*, Myxococcales*, Halobacteriovorax*; see Isnansetyo and Kamei 2009; Rosales et al. 2019; Li et al. 2022; Ricci et al. 2022; Speare et al. 2025) whereas CC7-SSB01 was differentially associated with taxa often linked to dysbiosis or disease (e.g., *Vibrio, Sphingobacteriales*; see Rosales et al. 2019; Speare et al. 2025; Ushijima et al. 2026). These taxa provide candidates for future culture-based, metagenomic, or experimental inoculation studies testing whether prokaryotic community members contribute causally to bleaching resistance.

### *Aiptasia* hosting thermally sensitive algae have more dissimilar prokaryotic communities

Unlike previous studies exploring coral and *Aiptasia* prokaryotic dynamics under thermal stress, we did not find strong evidence for increased community variance for the prokaryotic community of thermally sensitive CC7-SSB01 animals as a function of the duration under heat stress. We expected to see such trends in line with the Anna Karenina Principle, which argues that animals under abiotic stress have a more variable microbiome community due to the inability of the host and/or microbiome to regulate community composition (Zaneveld et al. 2017). This pattern has been widely reported for corals and more recently for *Aiptasia* bacterial communities, emerging as a hallmark of prokaryotic dysbiosis (Bourne et al. 2008; Wang et al. 2018; Ahmed et al. 2019; Greene et al. 2023; Glynn et al. 2025). Instead, via our ordination and network analyses, we observed that the prokaryotic microbiome of CC7-SSB01 animals was more dissimilar between any two pairs of samples, as compared to CC7-SSA01 animals, irrespective of the duration of heat stress. Thus, a more stochastic and potentially unstable prokaryotic community may represent a key characteristic of innately more thermally sensitive cnidarian holobionts. Replicating our heat stress assay with different *Aiptasia* clonal backgrounds and algal symbionts, alongside testing different stressors (e.g., nutrient, pH, salinity), will allow us to interrogate in a standardized fashion how general this observation is.

## Conclusion

In generating clonal *Aiptasia* lines associated with single algal symbiont strains, we show that algal symbionts not only impact host thermotolerance but may also fundamentally shape their animal’s prokaryotic microbiome. We also revealed potential taxa that may be indicators of both improved and reduced bleaching resilience. Finally, our results suggest that sustained community variance, as opposed to increased variance as a function of the duration under heat stress, may be a characteristic of more thermally sensitive cnidarian holobionts. Microorganisms play a central role in overall holobiont structure and thermotolerance across the tree of life (see McFall-Ngai et al. 2013). *Aiptasia* represents a tractable system for high-resolution studies on the structure and function of cnidarian holobionts, by allowing us to control for and manipulate the algal microbiome and distill the role of algal–prokaryotic interactions in coral bleaching resistance. In doing so, we will gain insights into how interactions between microbiome members have the ability to shape overall coral fitness, and in turn support some of the world’s most productive ecosystems.

## Author contributions

V.M.G. and R.D.H.B. designed the study. V.M.G. and E.C.L. performed the acute thermal stress assay and data collection. V.M.G. led the statistical analyses, with guidance from R.D.H.B. P.A.C. and R.D.H.B. jointly supervised the overall research project. V.M.G. wrote the manuscript with substantive contributions from R.D.H.B.

## Supplementary Material

icag086_Supplemental_File

## Data Availability

All scripts used in our analyses can be found at https://doi.org/10.5281/zenodo.20863659. All generated sequence data are available on the NCBI Sequence Read Archive (SRA) under NCBI BioProject PRJNA1436756.

## Notes

From the symposium “Host-microbial interactions across biological scales in a changing world” presented at the annual meeting of the Society for Integrative and Comparative Biology, January 3–7th, 2026.
