# Supplementary material for: Symbiont Identity Impacts Prokaryotic Microbiome Dynamics During Heat Stress in a Model System for Corals
Source: Integr Comp Biol. 2026 Jun 26;66:icag086. doi: 10.1093/icb/icag086 (PMC13367008; doi:10.1093/icb/icag086)
Supplement: icag086_Supplemental_File [file icag086_supplemental_file.docx]

**Supplementary Materials**

**Table S1**. **PCR amplification and barcoding conditions for heat stress samples.** All samples used the Earth Microbiome Project’s updated 515F (5’-GTGYCAGCMGCCGCGGTAA-3’; Parada et al. 2016) and 806R (5’-GGACTACNVGGGTWTCTAAT-3’; Apprill et al., 2015) primer pair (Ul-Hasan et al. 2019; <https://earthmicrobiome.org/protocols-and-standards/16s/>). Here, both 16S amplification and barcoding were performed in a single PCR step using the KAPA HiFi 2X Ready Mix. “Ind.” refers to the volume indicated under 1x reaction to be individually added to each PCR reaction.

| **16S amplification & barcoding one-step master mix reagent recipe** | | |
| --- | --- | --- |
|  | **1x** | **70x** |
| **Components** | **Volume (uL)** | |
| DNA input | 3 | Ind. |
| Forward primer 5uM | 2 | Ind. |
| Reverse primer 5uM | 2 | Ind. |
| 2X Kapa HiFi HotStart ready mix | 12.5 | 875 |
| H2O | 5.5 | 385 |
| TOTAL (uL) | 25 |  |

| **16S amplification & barcoding cycling conditions** | | |
| --- | --- | --- |
| **95°C** | 3min | |
| **95°C** | 30sec | x30 cycles |
| **58°C** | 30sec |  |
| **72°C** | 30sec |  |
| **72°C** | 5min | |
| **4°C** | Hold | |

**Table S2. PCR amplification and barcoding conditions for control samples.** All samples used the Earth Microbiome Project’s updated 515F (5’-GTGYCAGCMGCCGCGGTAA-3’; Parada et al. 2016) and 806R (5’-GGACTACNVGGGTWTCTAAT-3’; Apprill et al., 2015) primer pair (Ul-Hasan et al. 2019; <https://earthmicrobiome.org/protocols-and-standards/16s/>). Here, a two-step PCR approach was taken, where 16S amplification was done with the Phusion HF or GC Buffer and Phusion DNA polymerase, followed by a separate PCR barcoding step using the 2X Kapa HiFi HotStart ready mix. “Ind.” refers to the volume indicated under 1x reaction to be individually added to each PCR reaction.

| **16S amplification master mix reagent recipe** | | |
| --- | --- | --- |
|  | **1x** | **50x** |
| **Components** | **Volume (uL)** | |
| 5X Phusion HF or GC Buffer | 5 | 250 |
| 10mM dNTPs | 0.5 | 25 |
| 5uM Forward primer | 2.5 | 125 |
| 5uM Reverse primer | 2.5 | 125 |
| Phusion DNA polymerase | 0.25 | 12.5 |
| Nuclease Free Water | 12.25 | 612.5 |
| DNA template | 2 | Ind. |
| TOTAL (uL) | 25 |  |

| **16S amplification cycling conditions - 1st PCR** | | |
| --- | --- | --- |
| **98°C** | 30sec | |
| **98°C** | 10sec | x30 cycles |
| **58°C** | 30sec |  |
| **72°C** | 30sec |  |
| **72°C** | 5min | |
| **4°C** | Hold | |

| **16S barcoding master mix reagent recipe** | | |
| --- | --- | --- |
|  | **1x** | **50x** |
| **Components** | **Volume (uL)** | |
| 1st PCR product dil. 1:10 | 2.5 | Ind. |
| Forward primer 2uM | 2.5 | 5 |
| Reverse primer 2uM | 2.5 | 5 |
| 2X Kapa HiFi HotStart ready mix | 12.5 | 625 |
| H2O | 5 | 250 |
| TOTAL (uL) | 25 |  |

| **16S barcoding cycling conditions – 2nd PCR** | | |
| --- | --- | --- |
| **95°C** | 3min | |
| **95°C** | 30sec | x12 cycles |
| **55°C** | 30sec |  |
| **72°C** | 30sec |  |
| **72°C** | 5min | |
| **4°C** | Hold | |

**Table S3. Permutational multivariate analysis of variance (PERMANOVA) for *Aiptasia*’s prokaryotic 16S amplicon sequence variants (ASV) during our acute thermal stress assay.** Here, both heat stress and control samples are analyzed together, using Bray-Curtis distances and 999 permutations**.** The model has 16S ASVs as a function of the three-way interaction between treatment (control versus heat stress), time point, and line (CC7-SSA01 versus CC7-SSB01).

| **Variable** | **Degrees of freedom** | **Sum of squares** | **R^2^** | **F statistic** | **Pr (>F)** |
| --- | --- | --- | --- | --- | --- |
| Treatment | 1 | 0.36106 | 0.36344 | 82.4287 | 0.001 |
| Time | 5 | 0.05363 | 0.05399 | 2.4488 | 0.001 |
| Line | 1 | 0.16426 | 0.16534 | 37.5002 | 0.001 |
| Treatment x Time | 5 | 0.04945 | 0.04977 | 2.2577 | 0.004 |
| Treatment x Line | 1 | 0.12397 | 0.12479 | 28.3020 | 0.001 |
| Time x Line | 5 | 0.02783 | 0.02801 | 1.2707 | 0.190 |
| Treatment x Time x Line | 5 | 0.02490 | 0.02506 | 1.1368 | 0.287 |
| Residuals | 43 | 0.18835 | 0.18959 |  |  |
| Total | 66 | 0.9934 | 1.00000 |  |  |

**Table S4. Permutational multivariate analysis of variance (PERMANOVA) for *Aiptasia*’s prokaryotic 16S amplicon sequence variants (ASV) during our acute thermal stress assay, for only heat stress animals.** We used Bray-Curtis distances and 999 permutations**.** The model has 16S ASVs as a function of line (CC7-SSA01 versus CC7-SSB01) and time point.

| **Variable** | **Degrees of freedom** | **Sum of squares** | **R^2^** | **F statistic** | **Pr (>F)** |
| --- | --- | --- | --- | --- | --- |
| Line | 1 | 0.20370 | 0.56290 | 52.1688 | 0.001 |
| Time | 5 | 0.05851 | 0.16170 | 2.9972 | 0.006 |
| Line x Time | 5 | 0.02547 | 0.07039 | 1.3047 | 0.232 |
| Residuals | 19 | 0.07419 | 0.20501 |  |  |
| Total | 30 | 0.36187 | 1.00000 |  |  |

**Table S5. Post-hoc analysis of the pairwise permutational multivariate analysis of variance (PERMANOVA) across time points for CC7-SSA01 *Aiptasia*’s prokaryotic communities during our acute thermal stress assay.** We used Bray-Curtis distances, 999 permutations, and implemented Benjamini and Hochberg p-value corrections due to multiple comparisons. Time points are on the basis of hours, e.g. 0h = 0 hours.

| **0h vs 3h** | **Degrees of freedom** | **Sum of squares** | **R^2^** | **F statistic** | **Pr (>F)** |
| --- | --- | --- | --- | --- | --- |
| Time | 1 | 0.0025395 | 0.15488 | 0.5498 | 0.8 |
| Residual | 3 | 0.0138571 | 0.84512 |  |  |
| Total | 4 | 0.0163966 | 1.00000 |  |  |

| **0h vs 12h** | **Degrees of freedom** | **Sum of squares** | **R^2^** | **F statistic** | **Pr (>F)** |
| --- | --- | --- | --- | --- | --- |
| Time | 1 | 0.0025297 | 0.33024 | 1.4792 | 0.2 |
| Residual | 3 | 0.0051304 | 0.66976 |  |  |
| Total | 4 | 0.0076601 | 1.00000 |  |  |

| **0h vs 24h** | **Degrees of freedom** | **Sum of squares** | **R^2^** | **F statistic** | **Pr (>F)** |
| --- | --- | --- | --- | --- | --- |
| Time | 1 | 0.0046374 | 0.88159 | 7.4452 | 0.3333 |
| Residual | 1 | 0.0006229 | 0.11841 |  |  |
| Total | 2 | 0.0052603 | 1.00000 |  |  |

| **0h vs 48h** | **Degrees of freedom** | **Sum of squares** | **R^2^** | **F statistic** | **Pr (>F)** |
| --- | --- | --- | --- | --- | --- |
| Time | 1 | 0.0031219 | 0.66261 | 3.9279 | 0.3333 |
| Residual | 2 | 0.0015896 | 0.33739 |  |  |
| Total | 3 | 0.0047115 | 1.00000 |  |  |

| **0h vs 96h** | **Degrees of freedom** | **Sum of squares** | **R^2^** | **F statistic** | **Pr (>F)** |
| --- | --- | --- | --- | --- | --- |
| Time | 1 | 0.0161419 | 0.83336 | 10.002 | 0.3333 |
| Residual | 2 | 0.0032278 | 0.16664 |  |  |
| Total | 3 | 0.0193697 | 1.00000 |  |  |

| **3h vs 12h** | **Degrees of freedom** | **Sum of squares** | **R^2^** | **F statistic** | **Pr (>F)** |
| --- | --- | --- | --- | --- | --- |
| Time | 1 | 0.002787 | 0.13576 | 0.6283 | 1 |
| Residual | 4 | 0.017742 | 0.86424 |  |  |
| Total | 5 | 0.020529 | 1.00000 |  |  |

| **24h vs 3h** | **Degrees of freedom** | **Sum of squares** | **R^2^** | **F statistic** | **Pr (>F)** |
| --- | --- | --- | --- | --- | --- |
| Time | 1 | 0.0032496 | 0.19714 | 0.4911 | 0.75 |
| Residual | 2 | 0.0132342 | 0.80286 |  |  |
| Total | 3 | 0.0164838 | 1.00000 |  |  |

| **24h vs 12h** | **Degrees of freedom** | **Sum of squares** | **R^2^** | **F statistic** | **Pr (>F)** |
| --- | --- | --- | --- | --- | --- |
| Time | 1 | 0.0038667 | 0.46174 | 1.7157 | 0.25 |
| Residual | 2 | 0.0045076 | 0.53826 |  |  |
| Total | 3 | 0.0083743 | 1.00000 |  |  |

| **24h vs 48h** | **Degrees of freedom** | **Sum of squares** | **R^2^** | **F statistic** | **Pr (>F)** |
| --- | --- | --- | --- | --- | --- |
| Time | 1 | 0.0033083 | 0.77387 | 3.4221 | 0.3333 |
| Residual | 1 | 0.0009667 | 0.22613 |  |  |
| Total | 2 | 0.0042751 | 1.00000 |  |  |

| **24h vs 96h** | **Degrees of freedom** | **Sum of squares** | **R^2^** | **F statistic** | **Pr (>F)** |
| --- | --- | --- | --- | --- | --- |
| Time | 1 | 0.0066404 | 0.71824 | 2.5492 | 0.3333 |
| Residual | 1 | 0.0026049 | 0.28176 |  |  |
| Total | 2 | 0.0092453 | 1.00000 |  |  |

| **48h vs 3h** | **Degrees of freedom** | **Sum of squares** | **R^2^** | **F statistic** | **Pr (>F)** |
| --- | --- | --- | --- | --- | --- |
| Time | 1 | 0.0047291 | 0.24982 | 0.999 | 0.5 |
| Residual | 3 | 0.0142009 | 0.75018 |  |  |
| Total | 4 | 0.0189301 | 1.00000 |  |  |

| **48h vs 12h** | **Degrees of freedom** | **Sum of squares** | **R^2^** | **F statistic** | **Pr (>F)** |
| --- | --- | --- | --- | --- | --- |
| Time | 1 | 0.0032279 | 0.37093 | 1.7689 | 0.2 |
| Residual | 3 | 0.0054743 | 0.62907 |  |  |
| Total | 4 | 0.0087022 | 1.00000 |  |  |

| **48h vs 96h** | **Degrees of freedom** | **Sum of squares** | **R^2^** | **F statistic** | **Pr (>F)** |
| --- | --- | --- | --- | --- | --- |
| Time | 1 | 0.0097416 | 0.73172 | 5.4549 | 0.3333 |
| Residual | 2 | 0.0035717 | 0.26828 |  |  |
| Total | 3 | 0.0133133 | 1.00000 |  |  |

| **96h vs 3h** | **Degrees of freedom** | **Sum of squares** | **R^2^** | **F statistic** | **Pr (>F)** |
| --- | --- | --- | --- | --- | --- |
| Time | 1 | 0.012951 | 0.44985 | 2.453 | 0.2 |
| Residual | 3 | 0.015839 | 0.55015 |  |  |
| Total | 4 | 0.028790 | 1.00000 |  |  |

| **96h vs 12h** | **Degrees of freedom** | **Sum of squares** | **R^2^** | **F statistic** | **Pr (>F)** |
| --- | --- | --- | --- | --- | --- |
| Time | 1 | 0.0137619 | 0.65927 | 5.8047 | 0.1 |
| Residual | 3 | 0.0071125 | 0.34073 |  |  |
| Total | 4 | 0.0208744 | 1.00000 |  |  |

**Table S6. Post-hoc analysis of the pairwise permutational multivariate analysis of variance (PERMANOVA) across time points for CC7-SSB01 *Aiptasia*’s prokaryotic communities during our acute thermal stress assay.** We used Bray-Curtis distances, 999 permutations, and implemented Benjamini and Hochberg p-value corrections due to multiple comparisons. Time points are on the basis of hours, e.g. 0h = 0 hours.

| **0h vs 3h** | **Degrees of freedom** | **Sum of squares** | **R^2^** | **F statistic** | **Pr (>F)** |
| --- | --- | --- | --- | --- | --- |
| Time | 1 | 0.0025849 | 0.12183 | 0.5549 | 0.9 |
| Residual | 4 | 0.0186321 | 0.87817 |  |  |
| Total | 5 | 0.0212170 | 1.00000 |  |  |

| **0h vs 12h** | **Degrees of freedom** | **Sum of squares** | **R^2^** | **F statistic** | **Pr (>F)** |
| --- | --- | --- | --- | --- | --- |
| Time | 1 | 0.007464 | 0.37255 | 2.375 | 0.1 |
| Residual | 4 | 0.012571 | 0.62745 |  |  |
| Total | 5 | 0.020035 | 1.00000 |  |  |

| **0h vs 24h** | **Degrees of freedom** | **Sum of squares** | **R^2^** | **F statistic** | **Pr (>F)** |
| --- | --- | --- | --- | --- | --- |
| Time | 1 | 0.011728 | 0.4927 | 3.8849 | 0.1 |
| Residual | 4 | 0.012076 | 0.5073 |  |  |
| Total | 5 | 0.023804 | 1.0000 |  |  |

| **0h vs 48h** | **Degrees of freedom** | **Sum of squares** | **R^2^** | **F statistic** | **Pr (>F)** |
| --- | --- | --- | --- | --- | --- |
| Time | 1 | 0.0053852 | 0.26956 | 1.4761 | 0.2 |
| Residual | 4 | 0.0145928 | 0.73044 |  |  |
| Total | 5 | 0.0199780 | 1.00000 |  |  |

| **0h vs 96h** | **Degrees of freedom** | **Sum of squares** | **R^2^** | **F statistic** | **Pr (>F)** |
| --- | --- | --- | --- | --- | --- |
| Time | 1 | 0.019803 | 0.52259 | 4.3786 | 0.1 |
| Residual | 4 | 0.018091 | 0.47741 |  |  |
| Total | 5 | 0.037893 | 1.00000 |  |  |

| **3h vs 12h** | **Degrees of freedom** | **Sum of squares** | **R^2^** | **F statistic** | **Pr (>F)** |
| --- | --- | --- | --- | --- | --- |
| Time | 1 | 0.0061203 | 0.24032 | 1.2654 | 0.4 |
| Residual | 4 | 0.0193475 | 0.75968 |  |  |
| Total | 5 | 0.0254678 | 1.00000 |  |  |

| **24h vs 3h** | **Degrees of freedom** | **Sum of squares** | **R^2^** | **F statistic** | **Pr (>F)** |
| --- | --- | --- | --- | --- | --- |
| Time | 1 | 0.010431 | 0.35619 | 2.2131 | 0.1 |
| Residual | 4 | 0.018853 | 0.64381 |  |  |
| Total | 5 | 0.029283 | 1.00000 |  |  |

| **24h vs 12h** | **Degrees of freedom** | **Sum of squares** | **R^2^** | **F statistic** | **Pr (>F)** |
| --- | --- | --- | --- | --- | --- |
| Time | 1 | 0.0036358 | 0.22133 | 1.137 | 0.5 |
| Residual | 4 | 0.0127914 | 0.77867 |  |  |
| Total | 5 | 0.0164272 | 1.00000 |  |  |

| **24h vs 48h** | **Degrees of freedom** | **Sum of squares** | **R^2^** | **F statistic** | **Pr (>F)** |
| --- | --- | --- | --- | --- | --- |
| Time | 1 | 0.013944 | 0.48488 | 3.7652 | 0.1 |
| Residual | 4 | 0.014813 | 0.51512 |  |  |
| Total | 5 | 0.028757 | 1.00000 |  |  |

| **24h vs 96h** | **Degrees of freedom** | **Sum of squares** | **R^2^** | **F statistic** | **Pr (>F)** |
| --- | --- | --- | --- | --- | --- |
| Time | 1 | 0.011038 | 0.37608 | 2.4111 | 0.1 |
| Residual | 4 | 0.018311 | 0.62392 |  |  |
| Total | 5 | 0.029349 | 1.00000 |  |  |

| **48h vs 3h** | **Degrees of freedom** | **Sum of squares** | **R^2^** | **F statistic** | **Pr (>F)** |
| --- | --- | --- | --- | --- | --- |
| Time | 1 | 0.0057885 | 0.21314 | 1.0835 | 0.5 |
| Residual | 4 | 0.0213695 | 0.78686 |  |  |
| Total | 5 | 0.0271579 | 1.00000 |  |  |

| **48h vs 12h** | **Degrees of freedom** | **Sum of squares** | **R^2^** | **F statistic** | **Pr (>F)** |
| --- | --- | --- | --- | --- | --- |
| Time | 1 | 0.010878 | 0.41541 | 2.8424 | 0.1 |
| Residual | 4 | 0.015308 | 0.58459 |  |  |
| Total | 5 | 0.026186 | 1.00000 |  |  |

| **48h vs 96h** | **Degrees of freedom** | **Sum of squares** | **R^2^** | **F statistic** | **Pr (>F)** |
| --- | --- | --- | --- | --- | --- |
| Time | 1 | 0.015135 | 0.42085 | 2.9067 | 0.1 |
| Residual | 4 | 0.020828 | 0.57915 |  |  |
| Total | 5 | 0.035963 | 1.00000 |  |  |

| **96h vs 3h** | **Degrees of freedom** | **Sum of squares** | **R^2^** | **F statistic** | **Pr (>F)** |
| --- | --- | --- | --- | --- | --- |
| Time | 1 | 0.020129 | 0.44735 | 3.2378 | 0.1 |
| Residual | 4 | 0.024867 | 0.55265 |  |  |
| Total | 5 | 0.044996 | 1.00000 |  |  |

| **96h vs 12h** | **Degrees of freedom** | **Sum of squares** | **R^2^** | **F statistic** | **Pr (>F)** |
| --- | --- | --- | --- | --- | --- |
| Time | 1 | 0.014741 | 0.43942 | 3.1354 | 0.1 |
| Residual | 4 | 0.018806 | 0.56058 |  |  |
| Total | 5 | 0.033547 | 1.00000 |  |  |

**Table S7. Analysis of heat stressed CC7-SSA01 *Aiptasia*’s prokaryotic communities’ homogeneity of multivariate dispersions across time points during our acute thermal stress assay.** We used Bray-Curtis distances and 999 permutations. Our group dispersion results were run in an ANOVA to determine if the variance of each group’s distance from the centroid was statistically significant. Tukey’s Honest Significant Differences test was used for post-hoc comparisons at a 95% confidence level, where the p-values shown have been adjusted for multiple comparisons. Time points are on the basis of hours, e.g. 0h = 0 hours.

**Permutation test for homogeneity of multivariate dispersions**

|  | **Degrees of freedom** | **Sum of squares** | **Mean squared error** | **F statistic** | **Number of permutations** | **Pr (>F)** |
| --- | --- | --- | --- | --- | --- | --- |
| Groups | 5 | 0.0040080 | 0.00080161 | 1.0389 | 999 | 0.473 |
| Residuals | 7 | 0.0054014 | 0.00077162 |  |  |  |

**Average distance to centroid**

| **0h** | **3h** | **12h** | **24h** | **48h** | **96h** |
| --- | --- | --- | --- | --- | --- |
| 0.01765 | 0.06378 | 0.03849 | 0.00000 | 0.02199 | 0.03609 |

**ANOVA**

|  | **Degrees of freedom** | **Sum of squares** | **Mean squared error** | **F statistic** | **Pr (>F)** |
| --- | --- | --- | --- | --- | --- |
| Groups | 5 | 0.0046884 | 0.00093768 | 5.9987 | 0.01802 |
| Residual | 7 | 0.0010942 | 0.00015631 |  |  |

**Tukey’s Honest Significant Differences test**

| **Group** | **Differences** | **Lower** | **Upper** | **Adjusted p-value** |
| --- | --- | --- | --- | --- |
| 3h vs 0h | 0.046129120 | 0.00287935 | 0.089378890 | 0.0370327 |
| 12h vs 0h | 0.020845586 | -0.02240418 | 0.064095356 | 0.5061301 |
| 24h vs 0h | -0.017647552 | -0.07567321 | 0.040378103 | 0.8456083 |
| 48h vs 0h | 0.004338173 | -0.04303958 | 0.051715923 | 0.9990086 |
| 96h vs 0h | 0.018442166 | -0.02893558 | 0.065819915 | 0.6885321 |
| 12h vs 3h | -0.025283535 | -0.06396730 | 0.013400236 | 0.2477398 |
| 24h vs 3h | -0.063776672 | -0.11848378 | -0.009069560 | 0.0239426 |
| 48h vs 3h | -0.041790947 | -0.08504072 | 0.001458823 | 0.0583306 |
| 96h vs 3h | -0.027686955 | -0.07093672 | 0.015562815 | 0.2630789 |
| 24h vs 12h | -0.038493138 | -0.09320025 | 0.016213975 | 0.1973787 |
| 48h vs 12h | -0.016507412 | -0.05975718 | 0.026742358 | 0.7035648 |
| 96h vs 12h | -0.002403420 | -0.04565319 | 0.040846350 | 0.9999124 |
| 48h vs 24h | 0.021985726 | -0.03603993 | 0.080011381 | 0.7090506 |
| 96h vs 24h | 0.036089718 | -0.02193594 | 0.094115373 | 0.2851678 |
| 96h vs 48h | 0.014103992 | -0.03327376 | 0.061481741 | 0.8558691 |

**Table S8. Analysis of heat stressed CC7-SSB01 *Aiptasia*’s prokaryotic communities’ homogeneity of multivariate dispersions across time points during our acute thermal stress assay.** We used Bray-Curtis distances and 999 permutations. Our group dispersion results were run in an ANOVA to determine if the variance of each group’s distance from the centroid was statistically significant. Tukey’s Honest Significant Differences test was used for post-hoc comparisons at a 95% confidence level, where the p-values shown have been adjusted for multiple comparisons. Time points are on the basis of hours, e.g. 0h = 0 hours.

**Permutation test for homogeneity of multivariate dispersions**

|  | **Degrees of freedom** | **Sum of squares** | **Mean squared error** | **F statistic** | **Number of permutations** | **Pr (>F)** |
| --- | --- | --- | --- | --- | --- | --- |
| Groups | 5 | 0.0010830 | 0.00021659 | 0.6258 | 999 | 0.716 |
| Residuals | 12 | 0.0041535 | 0.00034612 |  |  |  |

**Average distance to centroid**

| **0h** | **3h** | **12h** | **24h** | **48h** | **96h** |
| --- | --- | --- | --- | --- | --- |
| 0.04398 | 0.06398 | 0.04692 | 0.04516 | 0.05365 | 0.06250 |

**ANOVA**

|  | **Degrees of freedom** | **Sum of squares** | **Mean squared error** | **F statistic** | **Pr (>F)** |
| --- | --- | --- | --- | --- | --- |
| Groups | 5 | 0.0011719 | 0.00023439 | 2.5811 | 0.08283 |
| Residual | 12 | 0.0010897 | 0.00009081 |  |  |

**Tukey’s Honest Significant Differences test**

| **Group** | **Differences** | **Lower** | **Upper** | **Adjusted p-value** |
| --- | --- | --- | --- | --- |
| 3h vs 0h | 0.020002613 | -0.006132248 | 0.046137474 | 0.1783977 |
| 12h vs 0h | 0.002946251 | -0.023188610 | 0.029081113 | 0.9987065 |
| 24h vs 0h | 0.001179665 | -0.024955196 | 0.027314527 | 0.9999854 |
| 48h vs 0h | 0.009677220 | -0.016457641 | 0.035812081 | 0.8080465 |
| 96h vs 0h | 0.018527977 | -0.007606884 | 0.044662838 | 0.2363312 |
| 12h vs 3h | -0.017056361 | -0.043191222 | 0.009078500 | 0.3080811 |
| 24h vs 3h | -0.018822947 | -0.044957808 | 0.007311914 | 0.2236581 |
| 48h vs 3h | -0.010325392 | -0.036460253 | 0.015809469 | 0.7658809 |
| 96h vs 3h | -0.001474636 | -0.027609497 | 0.024660225 | 0.9999559 |
| 24h vs 12h | -0.001766586 | -0.027901447 | 0.024368275 | 0.9998924 |
| 48h vs 12h | 0.006730969 | -0.019403892 | 0.032865830 | 0.9479968 |
| 96h vs 12h | 0.015581726 | -0.010553136 | 0.041716587 | 0.3942119 |
| 48h vs 24h | 0.008497555 | -0.017637306 | 0.034632416 | 0.8754492 |
| 96h vs 24h | 0.017348312 | -0.008786550 | 0.043483173 | 0.2927045 |
| 96h vs 48h | 0.008850757 | -0.017284104 | 0.034985618 | 0.8567068 |

**Table S9.** **Kruskal-Wallis test comparing algal cell densities across lines, grouped by time points.** Here, we compared our four possible treatment groups (i.e., CC7-SSA01 control, CC7-SSA01 heat stress, CC7-SSB01 control, CC7-SSB01 heat stress) across our four timepoints (i.e., 0, 24, 48, 96 hours). Post-hoc comparisons using Dunn’s test show adjusted p-values using Benjamini and Hochberg correction due to multiple comparisons. Groups are the various treatment groups.

| **Time (hours)** | **P-value** |
| --- | --- |
| 0 | 1.668642e-01 |
| 24 | 1.275083e-01 |
| 48 | 1.989429e-02 |
| 96 | 2.556069e-06 |

| **Time (hours)** | **Group 1** | **Group 2** | **Sample counts group 1** | **Sample counts group 2** | **Test statistic** | **p-value** | **Adjusted p-value** |
| --- | --- | --- | --- | --- | --- | --- | --- |
| 0 | SSA01_Control | SSA01_Heat | 6 | 18 | 2.0565 | 3.97E-02 | 1.91E-01 |
| 0 | SSA01_Control | SSB01_Control | 6 | 4 | 0.6405 | 5.22E-01 | 6.26E-01 |
| 0 | SSA01_Control | SSB01_Heat | 6 | 12 | 1.8534 | 6.38E-02 | 1.91E-01 |
| 0 | SSA01_Heat | SSB01_Control | 18 | 4 | -1.0059 | 3.14E-01 | 5.61E-01 |
| 0 | SSA01_Heat | SSB01_Heat | 18 | 12 | -0.1148 | 9.09E-01 | 9.09E-01 |
| 0 | SSB01_Control | SSB01_Heat | 4 | 12 | 0.889 | 3.74E-01 | 5.61E-01 |
| 24 | SSA01_Heat | SSB01_Heat | 18 | 12 | 1.524 | 1.28E-01 | 1.28E-01 |
| 48 | SSA01_Heat | SSB01_Heat | 18 | 12 | -2.3283 | 1.99E-02 | 1.99E-02 |
| 96 | SSA01_Control | SSA01_Heat | 6 | 18 | -2.5505 | 1.08E-02 | 1.29E-02 |
| 96 | SSA01_Control | SSB01_Control | 6 | 4 | 0.4086 | 6.83E-01 | 6.83E-01 |
| 96 | SSA01_Control | SSB01_Heat | 6 | 12 | -4.3625 | 1.29E-05 | 6.86E-05 |
| 96 | SSA01_Heat | SSB01_Control | 18 | 4 | 2.6522 | 8.00E-03 | 1.29E-02 |
| 96 | SSA01_Heat | SSB01_Heat | 18 | 12 | -2.6268 | 8.62E-03 | 1.29E-02 |
| 96 | SSB01_Control | SSB01_Heat | 4 | 12 | -4.2349 | 2.29E-05 | 6.86E-05 |

**Table S10.** **Kruskal-Wallis test comparing algal cell densities across time points, grouped by treatment groups.** Here, we compared our four timepoints (i.e., 0, 24, 48, 96 hours) across our four possible treatment groups (i.e., CC7-SSA01 control, CC7-SSA01 heat stress, CC7-SSB01 control, CC7-SSB01 heat stress). Post-hoc comparisons using Dunn’s test show adjusted p-values using Benjamini and Hochberg correction due to multiple comparisons. Pairwise group comparisons are across time points, e.g., 0 = 0 hours.

| **Treatment** | **P-value** |
| --- | --- |
| SSA01_Control | 2.623317e-01 |
| SSA01_Heat | 5.184874e-09 |
| SSB01_Control | 2.092134e-02 |
| SSB01_Heat | 1.553048e-08 |

| **Treatment** | **Group 1** | **Group 2** | **Sample counts group 1** | **Sample counts group 2** | **Test statistic** | **p-value** | **Adjusted p-value** |
| --- | --- | --- | --- | --- | --- | --- | --- |
| SSA01_Control | 0 | 96 | 6 | 6 | 1.1209 | 2.62E-01 | 2.62E-01 |
| SSA01_Heat | 0 | 24 | 18 | 18 | -1.7759 | 7.58E-02 | 9.09E-02 |
| SSA01_Heat | 0 | 48 | 18 | 18 | -4.826 | 1.39E-06 | 4.18E-06 |
| SSA01_Heat | 0 | 96 | 18 | 18 | -5.6303 | 1.80E-08 | 1.08E-07 |
| SSA01_Heat | 24 | 48 | 18 | 18 | -3.0501 | 2.29E-03 | 3.43E-03 |
| SSA01_Heat | 24 | 96 | 18 | 18 | -3.8544 | 1.16E-04 | 2.32E-04 |
| SSA01_Heat | 48 | 96 | 18 | 18 | -0.8043 | 4.21E-01 | 4.21E-01 |
| SSB01_Control | 0 | 96 | 4 | 4 | 2.3094 | 2.09E-02 | 2.09E-02 |
| SSB01_Heat | 0 | 24 | 12 | 12 | -0.175 | 8.61E-01 | 8.61E-01 |
| SSB01_Heat | 0 | 48 | 12 | 12 | -3.2951 | 9.84E-04 | 1.97E-03 |
| SSB01_Heat | 0 | 96 | 12 | 12 | -5.2781 | 1.31E-07 | 7.83E-07 |
| SSB01_Heat | 24 | 48 | 12 | 12 | -3.1202 | 1.81E-03 | 2.71E-03 |
| SSB01_Heat | 24 | 96 | 12 | 12 | -5.1031 | 3.34E-07 | 1.00E-06 |
| SSB01_Heat | 48 | 96 | 12 | 12 | -1.9829 | 4.74E-02 | 5.69E-02 |

**Table S11. Indicator species analysis comparing *Aiptasia*’s prokaryotic communities across lines during our acute thermal stress assay.** Only p < 0.05 are shown, which are corrected for multiple testing via the Benjamini and Hochberg p-value correction. Taxonomic information is shown down to the genus level, determined using the SILVA 16S database release v138.2 (Quast et al. 2013; Callahan 2024). Taxonomic levels for which no information could be ascertained are denoted with “NA.” Relative abundance of each taxon within each line (CC7-SSA01 vs CC7-SSB01) are shown as their own columns, respectively

| **Taxa** | **CC7-SSA01** | **CC7-SSB01** | Species indicator value | **P-value** | **Adjusted**  **p-value** | **Mean Relative Abundance CC7-SSA01** | **Mean Relative Abundance CC7-SSB01** |
| --- | --- | --- | --- | --- | --- | --- | --- |
| Bacteria Acidobacteriota Holophagae Acanthopleuribacterales *Acanthopleuribacteraceae Acanthopleuribacter* | 0 | 1 | 0.789443972 | 0.001 | 0.001802632 | 0.003358648 | 0.004972028 |
| Bacteria Actinomycetota Acidimicrobiia Microtrichales Microtrichaceae Sva0996 marine group | 0 | 1 | 0.838476454 | 0.001 | 0.001802632 | 0.000566477 | 0.001182474 |
| Bacteria Actinomycetota Actinobacteria Actinomycetales *Actinomycetaceae Actinomyces* | 1 | 0 | 0.754263123 | 0.001 | 0.001802632 | 0.000248929 | 0.000170437 |
| Bacteria Actinomycetota Actinobacteria Frankiales *Geodermatophilaceae Blastococcus* | 1 | 0 | 0.759195556 | 0.001 | 0.001802632 | 0.000233029 | 0.000154829 |
| Bacteria Actinomycetota Actinobacteria Micrococcales *Micrococcaceae Micrococcus* | 1 | 0 | 0.752339964 | 0.001 | 0.001802632 | 0.000258907 | 0.000179344 |
| Bacteria Actinomycetota Actinobacteria Mycobacteriales *Corynebacteriaceae Lawsonella* | 1 | 0 | 0.806485961 | 0.001 | 0.001802632 | 0.000311916 | 0.000152605 |
| Bacteria Actinomycetota Actinobacteria Propionibacteriales *Nocardioidaceae Nocardioides* | 1 | 0 | 0.758326566 | 0.001 | 0.001802632 | 0.000235267 | 0.000157149 |
| Bacteria Bacillota Bacilli Bacillales *Bacillaceae Metabacillus* | 1 | 0 | 0.755743957 | 0.001 | 0.001802632 | 0.000248049 | 0.000168315 |
| Bacteria Bacillota Bacilli Exiguobacterales *Exiguobacteraceae Exiguobacterium* | 1 | 0 | 0.758224767 | 0.001 | 0.001802632 | 0.000237052 | 0.00015844 |
| Bacteria Bacillota Bacilli Lactobacillales *Aerococcaceae Abiotrophia* | 1 | 0 | 0.757756128 | 0.001 | 0.001802632 | 0.000238002 | 0.00015953 |
| Bacteria Bacillota Bacilli Lactobacillales *Aerococcaceae Aerosphaera* | 1 | 0 | 0.810543499 | 0.001 | 0.001802632 | 0.000381083 | 0.000180523 |
| Bacteria Bacillota Bacilli Lactobacillales *Lactobacillaceae Lactobacillus* | 1 | 0 | 0.761911645 | 0.001 | 0.001802632 | 0.00022343 | 0.000145998 |
| Bacteria Bacillota Bacilli Lactobacillales *Streptococcaceae Streptococcus* | 1 | 0 | 0.719349959 | 0.001 | 0.001802632 | 0.000596999 | 0.000502036 |
| Bacteria Bacillota Clostridia Peptostreptococcales-Tissierellales *Caminicellaceae Wukongibacter* | 0 | 1 | 0.855127036 | 0.031 | 0.044239583 | 0.001050868 | 0.002496006 |
| Bacteria Bacteroidota Bacteroidia Chitinophagales NA NA | 1 | 0 | 0.730025713 | 0.035 | 0.048928571 | 0.008582686 | 0.006840308 |
| Bacteria Bacteroidota Bacteroidia Chitinophagales *Saprospiraceae Aureispira* | 1 | 0 | 0.710359561 | 0.002 | 0.003425 | 0.008059848 | 0.007138636 |
| Bacteria Bacteroidota Bacteroidia Chitinophagales *Saprospiraceae Flavilitoribacter* | 0 | 1 | 0.78096187 | 0.009 | 0.014091429 | 0.002628815 | 0.003732666 |
| Bacteria Bacteroidota Bacteroidia Chitinophagales *Saprospiraceae Lewinella* | 0 | 1 | 0.741113366 | 0.001 | 0.001802632 | 0.00508742 | 0.005557878 |
| Bacteria Bacteroidota Bacteroidia Chitinophagales *Saprospiraceae* NA | 0 | 1 | 0.750558565 | 0.001 | 0.001802632 | 0.009072047 | 0.010593308 |
| Bacteria Bacteroidota Bacteroidia Chitinophagales *Saprospiraceae Portibacter* | 0 | 1 | 0.729256794 | 0.003 | 0.004981818 | 0.006224509 | 0.006373725 |
| Bacteria Bacteroidota Bacteroidia Cytophagales *Cyclobacteriaceae* NA | 1 | 0 | 0.860582745 | 0.001 | 0.001802632 | 0.004193957 | 0.001367666 |
| Bacteria Bacteroidota Bacteroidia Cytophagales *Cyclobacteriaceae Roseivirga* | 1 | 0 | 0.734600971 | 0.003 | 0.004981818 | 0.013115285 | 0.010165862 |
| Bacteria Bacteroidota Bacteroidia Flavobacteriales *Crocinitomicaceae Crocinitomix* | 0 | 1 | 0.873624807 | 0.001 | 0.001802632 | 0.00049236 | 0.00138968 |
| Bacteria Bacteroidota Bacteroidia Flavobacteriales *Cryomorphaceae Owenweeksia* | 0 | 1 | 0.858480316 | 0.001 | 0.001802632 | 0.000901573 | 0.002217533 |
| Bacteria Bacteroidota Bacteroidia Flavobacteriales *Cryomorphaceae Vicingus* | 0 | 1 | 0.779231743 | 0.001 | 0.001802632 | 0.004087326 | 0.005682184 |
| Bacteria Bacteroidota Bacteroidia Flavobacteriales *Flavobacteriaceae Flagellimonas* | 1 | 0 | 0.729326103 | 0.001 | 0.001802632 | 0.007271516 | 0.005799237 |
| Bacteria Bacteroidota Bacteroidia Flavobacteriales *Flavobacteriaceae Kordia* | 1 | 0 | 0.791241551 | 0.001 | 0.001802632 | 0.006226362 | 0.00336873 |
| Bacteria Bacteroidota Bacteroidia Flavobacteriales *Flavobacteriaceae Mesoflavibacter* | 0 | 1 | 0.842448911 | 0.001 | 0.001802632 | 0.001717939 | 0.003691328 |
| Bacteria Bacteroidota Bacteroidia Flavobacteriales *Flavobacteriaceae Muricauda* | 0 | 1 | 0.753071151 | 0.001 | 0.001802632 | 0.002817989 | 0.003319114 |
| Bacteria Bacteroidota Bacteroidia Flavobacteriales *Flavobacteriaceae Pseudofulvibacter* | 0 | 1 | 0.783565606 | 0.001 | 0.001802632 | 0.004138581 | 0.005939371 |
| Bacteria Bacteroidota Bacteroidia Flavobacteriales *Flavobacteriaceae Pseudotenacibaculum* | 0 | 1 | 0.822304279 | 0.001 | 0.001802632 | 0.002085248 | 0.003863828 |
| Bacteria Bacteroidota Bacteroidia Flavobacteriales *Flavobacteriaceae Ulvibacterium* | 1 | 0 | 0.883893315 | 0.001 | 0.001802632 | 0.001328082 | 0.000358416 |
| Bacteria Bacteroidota Bacteroidia NA NA NA | 1 | 0 | 0.722961852 | 0.016 | 0.023956284 | 0.011416437 | 0.009436328 |
| Bacteria Bacteroidota Bacteroidia Sphingobacteriales NS11-12 marine group NA | 0 | 1 | 0.800382559 | 0.001 | 0.001802632 | 0.001174468 | 0.00186338 |
| Bacteria Bdellovibrionota Bacteriovoracia Bacteriovoracales *Bacteriovoracaceae Halobacteriovorax* | 1 | 0 | 0.732706913 | 0.001 | 0.001802632 | 0.009464946 | 0.007361083 |
| Bacteria Bdellovibrionota Bacteriovoracia Bacteriovoracales *Bacteriovoracaceae Peredibacter* | 1 | 0 | 0.751911587 | 0.001 | 0.001802632 | 0.009776946 | 0.006799894 |
| Bacteria Bdellovibrionota Bdellovibrionia Bdellovibrionales *Pseudobdellovibrionaceae* NA | 0 | 1 | 0.734004441 | 0.004 | 0.00660241 | 0.011896742 | 0.012575711 |
| Bacteria Bdellovibrionota Oligoflexia 053A03-B-DI-P58 NA NA | 1 | 0 | 0.760985938 | 0.001 | 0.001802632 | 0.00022656 | 0.000148888 |
| Bacteria Bdellovibrionota Oligoflexia Oligoflexales *Oligoflexaceae* NA | 1 | 0 | 0.718301949 | 0.015 | 0.022707182 | 0.013178413 | 0.011154783 |
| Bacteria Candidatus Eremiobacterota Eremiobacteria NA NA NA | 0 | 1 | 0.82313558 | 0.001 | 0.001802632 | 0.00427772 | 0.008005455 |
| Bacteria Candidatus Kapabacteria Kapabacteria Kapabacteriales NA NA | 0 | 1 | 0.836854126 | 0.034 | 0.048020619 | 0.000280823 | 0.000580568 |
| Bacteria Chlamydiota Chlamydiia Chlamydiales *Chlamydiaceae* NA | 0 | 1 | 0.817549406 | 0.016 | 0.023956284 | 0.000529254 | 0.000934923 |
| Bacteria Chlamydiota Chlamydiia *Chlamydiales* NA NA | 1 | 0 | 0.84215051 | 0.018 | 0.026659459 | 0.000486533 | 0.000186403 |
| *Bacteria Chloroflexota Anaerolineae Aggregatilineales Aggregatilineaceae NA* | 0 | 1 | 0.807704997 | 0.001 | 0.001802632 | 0.001984882 | 0.003321025 |
| Bacteria Chloroflexota Anaerolineae Ardenticatenales NA NA | 0 | 1 | 0.764092244 | 0.001 | 0.001802632 | 0.008532768 | 0.010704497 |
| Bacteria Chloroflexota Anaerolineae Caldilineales *Caldilineaceae* NA | 0 | 1 | 0.86620586 | 0.001 | 0.001802632 | 0.000630136 | 0.001650148 |
| Bacteria Chloroflexota Anaerolineae NA NA NA | 1 | 0 | 0.751371771 | 0.001 | 0.001802632 | 0.000266764 | 0.000185869 |
| Bacteria Cyanobacteriota Cyanobacteriia Limnotrichales *Limnotrichaceae Limnothrix* | 1 | 0 | 0.74179282 | 0.001 | 0.001802632 | 0.003317241 | 0.002471818 |
| Bacteria Cyanobacteriota Cyanobacteriia Phormidesmiales Nodosilineaceae *Halomicronema* TFEP1 | 0 | 1 | 0.746013169 | 0.001 | 0.001802632 | 0.00585082 | 0.006597033 |
| Bacteria Cyanobacteriota Cyanobacteriia Phormidesmiales *Nodosilineaceae* MBIC10086 | 1 | 0 | 0.825818145 | 0.001 | 0.001802632 | 0.010352617 | 0.004560754 |
| Bacteria Cyanobacteriota Cyanobacteriia Thermosynechococcales NA NA | 0 | 1 | 0.72519841 | 0.022 | 0.03206383 | 0.002679685 | 0.002669427 |
| Bacteria Cyanobacteriota Vampirivibrionia Obscuribacterales *Obscuribacteraceae* NA | 1 | 0 | 0.760724619 | 0.001 | 0.001802632 | 0.000227443 | 0.000149708 |
| Bacteria Deinococcota Deinococci Thermales *Thermaceae Thermus* | 1 | 0 | 0.751036813 | 0.001 | 0.001802632 | 0.00026707 | 0.000186459 |
| Bacteria Dependentiae Babeliae Babeliales *Vermiphilaceae* NA | 1 | 0 | 0.869002528 | 0.001 | 0.001802632 | 0.005130221 | 0.001524664 |
| Bacteria Hydrogenedentes Hydrogenedentia Hydrogenedentiales *Hydrogenedensaceae* NA | 0 | 1 | 0.841952596 | 0.001 | 0.001802632 | 0.003362205 | 0.00720992 |
| Bacteria Myxococcota Myxococcia Myxococcales *Myxococcaceae* P3OB-42 | 1 | 0 | 0.756733741 | 0.001 | 0.001802632 | 0.004607996 | 0.003112487 |
| Bacteria Myxococcota Polyangiia Nannocystales *Nannocystaceae Nannocystis* | 1 | 0 | 0.738682483 | 0.001 | 0.001802632 | 0.000342921 | 0.000257714 |
| Bacteria Myxococcota Polyangiia Polyangiales Eel-36e1D6 NA | 1 | 0 | 0.731128702 | 0.001 | 0.001802632 | 0.001535762 | 0.001219595 |
| Bacteria Myxococcota Polyangiia Polyangiales *Sandaracinaceae Sandaracinus* | 1 | 0 | 0.854850328 | 0.002 | 0.003425 | 0.001552022 | 0.000512012 |
| Bacteria NA NA NA NA NA | 1 | 0 | 0.768158626 | 0.001 | 0.001802632 | 0.008366387 | 0.00527877 |
| Bacteria Nitrospinota Nitrospinia Nitrospinales *Nitrospinaceae* LS-NOB | 1 | 0 | 0.740144001 | 0.001 | 0.001802632 | 0.000318974 | 0.000237656 |
| Bacteria PAUC34f NA NA NA NA | 1 | 0 | 0.852300284 | 0.001 | 0.001802632 | 0.001235953 | 0.000419873 |
| Bacteria Patescibacteria ABY1 NA NA NA | 1 | 0 | 0.749931872 | 0.001 | 0.001802632 | 0.00027338 | 0.000192138 |
| Bacteria Patescibacteria Gracilibacteria Candidatus Peregrinibacteria NA NA | 1 | 0 | 0.840793185 | 0.001 | 0.001802632 | 0.001535301 | 0.000600213 |
| Bacteria Patescibacteria Gracilibacteria NA NA NA | 0 | 1 | 0.780265313 | 0.002 | 0.003425 | 0.001798149 | 0.002511182 |
| Bacteria Patescibacteria Microgenomatia Candidatus Pacebacteria NA NA | 1 | 0 | 0.752689327 | 0.001 | 0.001802632 | 0.000261477 | 0.000180742 |
| Bacteria Planctomycetota BD7-11 NA NA NA | 0 | 1 | 0.794749241 | 0.007 | 0.011086705 | 0.002689403 | 0.004145496 |
| Bacteria Planctomycetota OM190 NA NA NA | 0 | 1 | 0.723846827 | 0.011 | 0.017028249 | 0.013756115 | 0.0136608 |
| Bacteria Planctomycetota Phycisphaerae Phycisphaerales *Phycisphaeraceae Algisphaera* | 1 | 0 | 0.743817923 | 0.001 | 0.001802632 | 0.000295485 | 0.000215409 |
| Bacteria Planctomycetota Phycisphaerae Phycisphaerales *Phycisphaeraceae* NA | 1 | 0 | 0.752589436 | 0.001 | 0.001802632 | 0.000258044 | 0.000178476 |
| Bacteria Planctomycetota Phycisphaerae Phycisphaerales *Phycisphaeraceae Phycisphaera* | 1 | 0 | 0.754496201 | 0.001 | 0.001802632 | 0.018147473 | 0.012419553 |
| Bacteria Planctomycetota Phycisphaerae Phycisphaerales *Phycisphaeraceae* SM1A02 | 0 | 1 | 0.808132802 | 0.001 | 0.001802632 | 0.003247915 | 0.005390958 |
| Bacteria Planctomycetota Phycisphaerae S-70 NA NA | 1 | 0 | 0.757576255 | 0.001 | 0.001802632 | 0.000239924 | 0.000160995 |
| Bacteria Planctomycetota Planctomycetes NA NA NA | 1 | 0 | 0.754654676 | 0.001 | 0.001802632 | 0.000498963 | 0.000340821 |
| Bacteria Planctomycetota Planctomycetes Pirellulales *Pirellulaceae Bremerella* | 0 | 1 | 0.74688193 | 0.001 | 0.001802632 | 0.00517182 | 0.00586369 |
| Bacteria Planctomycetota Planctomycetes Pirellulales *Pirellulaceae Mariniblastus* | 1 | 0 | 0.726294108 | 0.001 | 0.001802632 | 0.001510172 | 0.001232298 |
| Bacteria Planctomycetota Planctomycetes Pirellulales *Pirellulaceae* Pir4 lineage | 1 | 0 | 0.843214553 | 0.001 | 0.001802632 | 0.005800065 | 0.002126089 |
| Bacteria Planctomycetota Planctomycetes Pirellulales *Pirellulaceae Pirellula* | 0 | 1 | 0.741725259 | 0.001 | 0.001802632 | 0.009984063 | 0.010956881 |
| Bacteria Planctomycetota Planctomycetes Pirellulales *Pirellulaceae Rhodopirellula* | 1 | 0 | 0.872214245 | 0.001 | 0.001802632 | 0.000496966 | 0.000152602 |
| Bacteria Planctomycetota Planctomycetes Planctomycetales *Gimesiaceae Gimesia* | 1 | 0 | 0.837869005 | 0.001 | 0.001802632 | 0.003201903 | 0.001216669 |
| Bacteria Planctomycetota Planctomycetes Planctomycetales *Gimesiaceae* NA | 1 | 0 | 0.763260041 | 0.03 | 0.043036649 | 0.004352783 | 0.002802341 |
| Bacteria Planctomycetota Planctomycetes Planctomycetales NA NA | 1 | 0 | 0.8140121 | 0.025 | 0.036243386 | 0.001244313 | 0.000563686 |
| Bacteria Planctomycetota Planctomycetes Planctomycetales *Rubinisphaeraceae Fuerstia* | 0 | 1 | 0.848058198 | 0.001 | 0.001802632 | 0.0013106 | 0.002953492 |
| Bacteria Planctomycetota Planctomycetes Planctomycetales *Rubinisphaeraceae* NA | 0 | 1 | 0.800152657 | 0.001 | 0.001802632 | 0.002725135 | 0.004321941 |
| Bacteria Planctomycetota Planctomycetes Planctomycetales *Rubinisphaeraceae Rubinisphaera* | 0 | 1 | 0.810233633 | 0.001 | 0.001802632 | 0.002374083 | 0.004059771 |
| Bacteria Pseudomonadota Alphaproteobacteria Caulobacterales *Hyphomonadaceae Algimonas* | 0 | 1 | 0.847183577 | 0.001 | 0.001802632 | 0.002346186 | 0.005302227 |
| Bacteria Pseudomonadota Alphaproteobacteria Caulobacterales *Hyphomonadaceae Maricaulis* | 1 | 0 | 0.711171779 | 0.019 | 0.027989247 | 0.009419074 | 0.008304107 |
| Bacteria Pseudomonadota Alphaproteobacteria Caulobacterales *Hyphomonadaceae Marinicauda* | 1 | 0 | 0.75996009 | 0.001 | 0.001802632 | 0.000230356 | 0.000152338 |
| Bacteria Pseudomonadota Alphaproteobacteria Caulobacterales *Hyphomonadaceae Oceanicaulis* | 1 | 0 | 0.713005214 | 0.006 | 0.009670588 | 0.011619188 | 0.010142981 |
| Bacteria Pseudomonadota Alphaproteobacteria Caulobacterales *Hyphomonadaceae Ponticaulis* | 1 | 0 | 0.730592597 | 0.001 | 0.001802632 | 0.000406021 | 0.000319954 |
| Bacteria Pseudomonadota Alphaproteobacteria Caulobacterales *Parvularculaceae* NA | 0 | 1 | 0.769744244 | 0.001 | 0.001802632 | 0.002916936 | 0.00383043 |
| Bacteria Pseudomonadota Alphaproteobacteria Defluviicoccales NA NA | 1 | 0 | 0.754357487 | 0.001 | 0.001802632 | 0.000245217 | 0.000167799 |
| Bacteria Pseudomonadota Alphaproteobacteria Hyphomicrobiales *Beijerinckiaceae Methylorubrum* | 1 | 0 | 0.760223762 | 0.001 | 0.001802632 | 0.000229407 | 0.000151466 |
| Bacteria Pseudomonadota Alphaproteobacteria Hyphomicrobiales *Devosiaceae Cucumibacter* | 1 | 0 | 0.738759846 | 0.001 | 0.001802632 | 0.000335082 | 0.000251707 |
| Bacteria Pseudomonadota Alphaproteobacteria Hyphomicrobiales *Devosiaceae Devosia* | 1 | 0 | 0.753638755 | 0.001 | 0.001802632 | 0.000512743 | 0.000352396 |
| Bacteria Pseudomonadota Alphaproteobacteria Hyphomicrobiales *Devosiaceae Maritalea* | 0 | 1 | 0.710750738 | 0.001 | 0.001802632 | 0.011783805 | 0.010843034 |
| Bacteria Pseudomonadota Alphaproteobacteria Hyphomicrobiales *Methyloligellaceae* NA | 1 | 0 | 0.759547599 | 0.001 | 0.001802632 | 0.00023251 | 0.000154151 |
| Bacteria Pseudomonadota Alphaproteobacteria Hyphomicrobiales *Rhizobiaceae Ahrensia* | 0 | 1 | 0.773543569 | 0.002 | 0.003425 | 0.000921343 | 0.0012244 |
| Bacteria Pseudomonadota Alphaproteobacteria Hyphomicrobiales *Rhizobiaceae Cohaesibacter* | 0 | 1 | 0.767093243 | 0.001 | 0.001802632 | 0.006744313 | 0.008641895 |
| Bacteria Pseudomonadota Alphaproteobacteria Hyphomicrobiales *Rhizobiaceae* NA | 0 | 1 | 0.78424891 | 0.001 | 0.001802632 | 0.004424877 | 0.006324174 |
| Bacteria Pseudomonadota Alphaproteobacteria Hyphomicrobiales *Rhizobiaceae Pseudahrensia* | 0 | 1 | 0.744456675 | 0.001 | 0.001802632 | 0.007609316 | 0.008499127 |
| Bacteria Pseudomonadota Alphaproteobacteria Hyphomicrobiales *Stappiaceae* NA | 1 | 0 | 0.763039946 | 0.001 | 0.001802632 | 0.00295577 | 0.001916054 |
| Bacteria Pseudomonadota Alphaproteobacteria Hyphomicrobiales *Stappiaceae Roseibium* | 0 | 1 | 0.759072301 | 0.001 | 0.001802632 | 0.01531862 | 0.018699101 |
| Bacteria Pseudomonadota Alphaproteobacteria Hyphomicrobiales *Stappiaceae Stappia* | 1 | 0 | 0.724456302 | 0.001 | 0.001802632 | 0.000523983 | 0.000427875 |
| Bacteria Pseudomonadota Alphaproteobacteria Hyphomicrobiales *Xanthobacteraceae Bradyrhizobium* | 1 | 0 | 0.755109332 | 0.001 | 0.001802632 | 0.000245366 | 0.000167137 |
| Bacteria Pseudomonadota Alphaproteobacteria Kiloniellales *Kiloniellaceae Kiloniella* | 0 | 1 | 0.821919692 | 0.001 | 0.001802632 | 0.001739508 | 0.003238038 |
| Bacteria Pseudomonadota Alphaproteobacteria Kiloniellales *Kiloniellaceae* NA | 1 | 0 | 0.793424053 | 0.003 | 0.004981818 | 0.002084485 | 0.001112593 |
| Bacteria Pseudomonadota Alphaproteobacteria Kiloniellales *Kiloniellaceae Tagaea* | 0 | 1 | 0.746772554 | 0.021 | 0.030770053 | 0.00378607 | 0.004253198 |
| Bacteria Pseudomonadota Alphaproteobacteria Kordiimonadales *Kordiimonadaceae Kordiimonas* | 0 | 1 | 0.770110875 | 0.001 | 0.001802632 | 0.004004218 | 0.005249879 |
| Bacteria Pseudomonadota Alphaproteobacteria Micavibrionales *Micavibrionaceae* NA | 0 | 1 | 0.763395489 | 0.001 | 0.001802632 | 0.015158229 | 0.019045871 |
| Bacteria Pseudomonadota Alphaproteobacteria Micavibrionales NA NA | 0 | 1 | 0.774057139 | 0.001 | 0.001802632 | 0.001859442 | 0.002464273 |
| Bacteria Pseudomonadota Alphaproteobacteria NA NA NA | 0 | 1 | 0.753980568 | 0.001 | 0.001802632 | 0.013492895 | 0.015965808 |
| Bacteria Pseudomonadota Alphaproteobacteria NRL2 NA NA | 0 | 1 | 0.744206679 | 0.001 | 0.001802632 | 0.006083228 | 0.006773614 |
| Bacteria Pseudomonadota Alphaproteobacteria Paracaedibacterales Paracaedibacteraceae *Candidatus Captivus* | 1 | 0 | 0.822054288 | 0.035 | 0.048928571 | 0.001376697 | 0.000597311 |
| Bacteria Pseudomonadota Alphaproteobacteria Parvibaculales *Parvibaculaceae* Mf105b01 | 0 | 1 | 0.889338671 | 0.001 | 0.001802632 | 0.000551449 | 0.001847075 |
| Bacteria Pseudomonadota Alphaproteobacteria Parvibaculales *Parvibaculaceae Tepidicaulis* | 1 | 0 | 0.756256844 | 0.001 | 0.001802632 | 0.000244112 | 0.000165128 |
| Bacteria Pseudomonadota Alphaproteobacteria Rhodobacterales *Paracoccaceae Actibacterium* | 1 | 0 | 0.711468453 | 0.001 | 0.001802632 | 0.001170364 | 0.001029482 |
| Bacteria Pseudomonadota Alphaproteobacteria Rhodobacterales *Paracoccaceae Aestuariicoccus* | 0 | 1 | 0.869559674 | 0.001 | 0.001802632 | 0.000797439 | 0.002177111 |
| Bacteria Pseudomonadota Alphaproteobacteria Rhodobacterales *Paracoccaceae Aliiroseovarius* | 0 | 1 | 0.852145986 | 0.001 | 0.001802632 | 0.000794993 | 0.001849286 |
| Bacteria Pseudomonadota Alphaproteobacteria Rhodobacterales *Paracoccaceae Amaricoccus* | 1 | 0 | 0.714562144 | 0.001 | 0.001802632 | 0.000781407 | 0.000675349 |
| Bacteria Pseudomonadota Alphaproteobacteria Rhodobacterales *Paracoccaceae Cognatishimia* | 0 | 1 | 0.716524233 | 0.001 | 0.001802632 | 0.01502556 | 0.014298493 |
| Bacteria Pseudomonadota Alphaproteobacteria Rhodobacterales *Paracoccaceae Litorimicrobium* | 0 | 1 | 0.828441057 | 0.001 | 0.001802632 | 0.002516488 | 0.004850549 |
| Bacteria Pseudomonadota Alphaproteobacteria Rhodobacterales *Paracoccaceae Maritimibacter* | 1 | 0 | 0.711220908 | 0.001 | 0.001802632 | 0.001213557 | 0.001068977 |
| Bacteria Pseudomonadota Alphaproteobacteria Rhodobacterales *Paracoccaceae Marivita* | 0 | 1 | 0.794268098 | 0.002 | 0.003425 | 0.001087762 | 0.001655209 |
| Bacteria Pseudomonadota Alphaproteobacteria Rhodobacterales *Paracoccaceae* NA | 0 | 1 | 0.745738062 | 0.001 | 0.001802632 | 0.027178852 | 0.030509052 |
| Bacteria Pseudomonadota Alphaproteobacteria Rhodobacterales *Paracoccaceae Nautella* | 0 | 1 | 0.765914066 | 0.001 | 0.001802632 | 0.003340502 | 0.004261134 |
| Bacteria Pseudomonadota Alphaproteobacteria Rhodobacterales *Paracoccaceae Pararhodobacter* | 1 | 0 | 0.755669783 | 0.001 | 0.001802632 | 0.000246971 | 0.000167658 |
| Bacteria Pseudomonadota Alphaproteobacteria Rhodobacterales *Paracoccaceae Pelagibaca* | 0 | 1 | 0.80416258 | 0.001 | 0.001802632 | 0.000933207 | 0.001511458 |
| Bacteria Pseudomonadota Alphaproteobacteria Rhodobacterales *Paracoccaceae Phaeobacter* | 0 | 1 | 0.878473124 | 0.003 | 0.004981818 | 0.000498123 | 0.001451704 |
| Bacteria Pseudomonadota Alphaproteobacteria Rhodobacterales *Paracoccaceae Pseudooceanicola* | 0 | 1 | 0.800227924 | 0.001 | 0.001802632 | 0.002322111 | 0.003677063 |
| Bacteria Pseudomonadota Alphaproteobacteria Rhodobacterales *Paracoccaceae Shimia* | 1 | 0 | 0.724203728 | 0.002 | 0.003425 | 0.009832747 | 0.00804205 |
| Bacteria Pseudomonadota Alphaproteobacteria Rhodobacterales *Paracoccaceae Silicimonas* | 1 | 0 | 0.824058576 | 0.002 | 0.003425 | 0.003023904 | 0.001312526 |
| Bacteria Pseudomonadota Alphaproteobacteria Rhodobacterales *Paracoccaceae Thalassococcus* | 1 | 0 | 0.707118279 | 0.001 | 0.001802632 | 0.006180255 | 0.005572 |
| Bacteria Pseudomonadota Alphaproteobacteria Rhodobacterales *Paracoccaceae Tritonibacter* | 0 | 1 | 0.805094983 | 0.001 | 0.001802632 | 0.001835626 | 0.00303301 |
| Bacteria Pseudomonadota Alphaproteobacteria Rhodospirillales NA NA | 0 | 1 | 0.848547014 | 0.001 | 0.001802632 | 0.001308349 | 0.0029909 |
| Bacteria Pseudomonadota Alphaproteobacteria Rhodospirillales *Rhodospirillaceae* NA | 1 | 0 | 0.773968176 | 0.001 | 0.001802632 | 0.00298515 | 0.001782984 |
| Bacteria Pseudomonadota Alphaproteobacteria Rhodospirillales *Terasakiellaceae Terasakiella* | 0 | 1 | 0.746018463 | 0.001 | 0.001802632 | 0.01033738 | 0.01168234 |
| Bacteria Pseudomonadota Alphaproteobacteria Rickettsiales AB1 NA | 0 | 1 | 0.75276145 | 0.001 | 0.001802632 | 0.005760927 | 0.006795033 |
| Bacteria Pseudomonadota Alphaproteobacteria Rickettsiales SM2D12 NA | 1 | 0 | 0.720141923 | 0.001 | 0.001802632 | 0.001254714 | 0.00105035 |
| Bacteria Pseudomonadota Alphaproteobacteria Sneathiellales *Sneathiellaceae Sneathiella* | 0 | 1 | 0.752921003 | 0.001 | 0.001802632 | 0.006205611 | 0.007292009 |
| Bacteria Pseudomonadota Alphaproteobacteria Sphingomonadales *Sphingomonadaceae* NA | 0 | 1 | 0.834055899 | 0.001 | 0.001802632 | 0.00070795 | 0.001447026 |
| Bacteria Pseudomonadota Alphaproteobacteria Sphingomonadales *Sphingomonadaceae Parasphingorhabdus* | 0 | 1 | 0.86543725 | 0.001 | 0.001802632 | 0.000986853 | 0.002593665 |
| Bacteria Pseudomonadota Alphaproteobacteria Sphingomonadales *Sphingomonadaceae Sphingobium* | 1 | 0 | 0.749676764 | 0.001 | 0.001802632 | 0.0002575 | 0.000181254 |
| Bacteria Pseudomonadota Alphaproteobacteria Sphingomonadales *Sphingomonadaceae Sphingomonas* | 1 | 0 | 0.721927525 | 0.001 | 0.001802632 | 0.001162528 | 0.000963241 |
| Bacteria Pseudomonadota Alphaproteobacteria Thalassobaculales NA NA | 1 | 0 | 0.749725572 | 0.001 | 0.001802632 | 0.000266212 | 0.000187332 |
| Bacteria Pseudomonadota Alphaproteobacteria Thalassobaculales *Nisaeaceae* OM75 clade | 1 | 0 | 0.732885035 | 0.007 | 0.011086705 | 0.002911214 | 0.002268428 |
| Bacteria Pseudomonadota Alphaproteobacteria Thalassobaculales *Thalassobaculaceae Thalassobaculum* | 1 | 0 | 0.749709632 | 0.001 | 0.001802632 | 0.010886583 | 0.007693401 |
| Bacteria Pseudomonadota Gammaproteobacteria Arenicellales *Arenicellaceae* HTCC5015 | 1 | 0 | 0.885939904 | 0.001 | 0.001802632 | 0.003959752 | 0.000996268 |
| Bacteria Pseudomonadota Gammaproteobacteria Burkholderiales Burkholderiaceae *Burkholderia-Caballeronia-Paraburkholderia* | 1 | 0 | 0.862445247 | 0.001 | 0.001802632 | 0.001854273 | 0.000565933 |
| Bacteria Pseudomonadota Gammaproteobacteria Burkholderiales *Comamonadaceae Aquabacterium* | 1 | 0 | 0.758838951 | 0.001 | 0.001802632 | 0.00023315 | 0.000155247 |
| Bacteria Pseudomonadota Gammaproteobacteria Burkholderiales *Comamonadaceae* NA | 1 | 0 | 0.754178173 | 0.001 | 0.001802632 | 0.000248778 | 0.000170422 |
| Bacteria Pseudomonadota Gammaproteobacteria Burkholderiales *Comamonadaceae Ottowia* | 1 | 0 | 0.761256675 | 0.001 | 0.001802632 | 0.000225628 | 0.000148029 |
| Bacteria Pseudomonadota Gammaproteobacteria Burkholderiales *Oxalobacteraceae Massilia* | 1 | 0 | 0.756231652 | 0.001 | 0.001802632 | 0.000242152 | 0.000163826 |
| Bacteria Pseudomonadota Gammaproteobacteria Enterobacterales *Alteromonadaceae Aestuariibacter* | 1 | 0 | 0.757079572 | 0.001 | 0.001802632 | 0.001840035 | 0.001243172 |
| Bacteria Pseudomonadota Gammaproteobacteria Enterobacterales *Alteromonadaceae* NA | 0 | 1 | 0.782289343 | 0.001 | 0.001802632 | 0.009426901 | 0.013427215 |
| Bacteria Pseudomonadota Gammaproteobacteria Enterobacterales *Alteromonadaceae Neptunicella* | 1 | 0 | 0.76122455 | 0.001 | 0.001802632 | 0.000226104 | 0.00014837 |
| Bacteria Pseudomonadota Gammaproteobacteria Enterobacterales *Alteromonadaceae Planctobacterium* | 0 | 1 | 0.72367839 | 0.001 | 0.001802632 | 0.007895847 | 0.007812503 |
| Bacteria Pseudomonadota Gammaproteobacteria Enterobacterales *Colwelliaceae Thalassotalea* | 1 | 0 | 0.707647746 | 0.001 | 0.001802632 | 0.002672921 | 0.002402646 |
| Bacteria Pseudomonadota Gammaproteobacteria Enterobacterales *Enterobacteriaceae Klebsiella* | 1 | 0 | 0.740000822 | 0.001 | 0.001802632 | 0.00032574 | 0.000242902 |
| Bacteria Pseudomonadota Gammaproteobacteria Enterobacterales *Idiomarinaceae Idiomarina* | 1 | 0 | 0.808521901 | 0.033 | 0.046849741 | 0.001215183 | 0.000568413 |
| Bacteria Pseudomonadota Gammaproteobacteria Enterobacterales NA NA | 1 | 0 | 0.763155282 | 0.001 | 0.001802632 | 0.000219099 | 0.000142075 |
| Bacteria Pseudomonadota Gammaproteobacteria Enterobacterales *Vibrionaceae Photobacterium* | 0 | 1 | 0.794975425 | 0.001 | 0.001802632 | 0.003480961 | 0.005329392 |
| Bacteria Pseudomonadota Gammaproteobacteria Enterobacterales *Vibrionaceae Vibrio* | 0 | 1 | 0.735581167 | 0.001 | 0.001802632 | 0.020959838 | 0.022201965 |
| Bacteria Pseudomonadota Gammaproteobacteria Francisellales *Francisellaceae Allofrancisella* | 1 | 0 | 0.909101144 | 0.001 | 0.001802632 | 0.001239567 | 0.000247499 |
| Bacteria Pseudomonadota Gammaproteobacteria Francisellales *Francisellaceae* NA | 0 | 1 | 0.750001706 | 0.001 | 0.001802632 | 0.001952746 | 0.002261975 |
| Bacteria Pseudomonadota Gammaproteobacteria JTB23 NA NA | 0 | 1 | 0.785735003 | 0.001 | 0.001802632 | 0.002825487 | 0.004110437 |
| Bacteria Pseudomonadota Gammaproteobacteria Legionellales *Legionellaceae Legionella* | 0 | 1 | 0.87778814 | 0.005 | 0.008203593 | 0.000300496 | 0.000895889 |
| Bacteria Pseudomonadota Gammaproteobacteria Legionellales *Legionellaceae* NA | 1 | 0 | 0.832324283 | 0.001 | 0.001802632 | 0.008796541 | 0.003538579 |
| Bacteria Pseudomonadota Gammaproteobacteria Lysobacterales *Lysobacteraceae Stenotrophomonas* | 1 | 0 | 0.727634908 | 0.009 | 0.014091429 | 0.001137449 | 0.000915566 |
| Bacteria Pseudomonadota Gammaproteobacteria Nitrosococcales *Nitrosococcaceae* Cm1-21 | 0 | 1 | 0.841796893 | 0.001 | 0.001802632 | 0.001766603 | 0.003791848 |
| Bacteria Pseudomonadota Gammaproteobacteria Pseudomonadales *Halieaceae* NA | 1 | 0 | 0.782739898 | 0.002 | 0.003425 | 0.004254165 | 0.002431835 |
| Bacteria Pseudomonadota Gammaproteobacteria Pseudomonadales KI89A clade NA | 1 | 0 | 0.757395555 | 0.012 | 0.018368715 | 0.005344242 | 0.00363999 |
| Bacteria Pseudomonadota Gammaproteobacteria Pseudomonadales *Marinobacteraceae Marinobacter* | 0 | 1 | 0.756350578 | 0.006 | 0.009670588 | 0.005869114 | 0.007065444 |
| Bacteria Pseudomonadota Gammaproteobacteria Pseudomonadales *Moraxellaceae Enhydrobacter* | 1 | 0 | 0.73083002 | 0.001 | 0.001802632 | 0.000361365 | 0.000284368 |
| Bacteria Pseudomonadota Gammaproteobacteria Pseudomonadales *Nitrincolaceae Neptuniibacter* | 0 | 1 | 0.792344838 | 0.001 | 0.001802632 | 0.002027772 | 0.003073428 |
| Bacteria Pseudomonadota Gammaproteobacteria Pseudomonadales OM182 clade NA | 0 | 1 | 0.798086649 | 0.001 | 0.001802632 | 0.003037063 | 0.004784011 |
| Bacteria Pseudomonadota Gammaproteobacteria Pseudomonadales *Oceanospirillaceae Oceanospirillum* | 0 | 1 | 0.730139605 | 0.017 | 0.025315217 | 0.016263291 | 0.01662564 |
| Bacteria Pseudomonadota Gammaproteobacteria Pseudomonadales P13-46 NA | 0 | 1 | 0.803254468 | 0.001 | 0.001802632 | 0.00261661 | 0.004267163 |
| Bacteria Pseudomonadota Gammaproteobacteria Pseudomonadales *Pseudomonadaceae Pseudomonas* | 1 | 0 | 0.745349811 | 0.014 | 0.021311111 | 0.006074292 | 0.004411537 |
| Bacteria Pseudomonadota Gammaproteobacteria Pseudomonadales *Saccharospirillaceae Bacterioplanes* | 0 | 1 | 0.774787744 | 0.001 | 0.001802632 | 0.003328669 | 0.004493405 |
| Bacteria Pseudomonadota Gammaproteobacteria Pseudomonadales *Spongiibacteraceae* BD1-7 clade | 0 | 1 | 0.784307751 | 0.001 | 0.001802632 | 0.002702859 | 0.003864235 |
| Bacteria Pseudomonadota Gammaproteobacteria S1-3-65 NA NA | 0 | 1 | 0.881260635 | 0.003 | 0.004981818 | 0.000365573 | 0.001093211 |
| Bacteria Pseudomonadota Gammaproteobacteria Salinisphaerales *Algiphilaceae Algiphilus* | 0 | 1 | 0.837445392 | 0.001 | 0.001802632 | 0.000743874 | 0.001549345 |
| Bacteria Pseudomonadota Gammaproteobacteria Salinisphaerales *Nevskiaceae Oceanococcus* | 0 | 1 | 0.778246198 | 0.012 | 0.018368715 | 0.001176278 | 0.001629496 |
| Bacteria Pseudomonadota NA NA NA NA | 1 | 0 | 0.753770696 | 0.001 | 0.001802632 | 0.000511018 | 0.00035093 |
| Bacteria Rhodothermota Rhodothermia Rhodothermales *Rhodothermaceae* NA | 0 | 1 | 0.758670953 | 0.001 | 0.001802632 | 0.00698005 | 0.008440247 |
| Bacteria SAR324 clade(Marine group B) Incertae Sedis Incertae Sedis Incertae Sedis Candidatus *Nitrosoarchaeum* | 0 | 1 | 0.857570014 | 0.001 | 0.001802632 | 0.001092574 | 0.002719926 |
| Bacteria Spirochaetota Leptospirae Leptospirales *Leptospiraceae* NA | 0 | 1 | 0.793173983 | 0.001 | 0.001802632 | 0.002594259 | 0.003930342 |
| Bacteria Sumerlaeota Sumerlaeia NA NA NA | 1 | 0 | 0.753209511 | 0.001 | 0.001802632 | 0.000259595 | 0.000178878 |
| Bacteria Thermodesulfobacteriota Desulfuromonadia Bradymonadales Incertae Sedis SKP-2 | 1 | 0 | 0.856345088 | 0.007 | 0.011086705 | 0.001367837 | 0.000446209 |
| Bacteria Thermodesulfobacteriota Desulfuromonadia PB19 NA NA | 0 | 1 | 0.7934982 | 0.001 | 0.001802632 | 0.004133706 | 0.006288019 |
| Bacteria Verrucomicrobiota Verrucomicrobiia Opitutales *Opitutaceae Diplosphaera* | 0 | 1 | 0.811363981 | 0.001 | 0.001802632 | 0.001839248 | 0.003152881 |
| Bacteria Verrucomicrobiota Verrucomicrobiia Opitutales *Puniceicoccaceae* NA | 0 | 1 | 0.851764183 | 0.027 | 0.038936842 | 0.000293607 | 0.000706043 |
| Bacteria Verrucomicrobiota Verrucomicrobiia Pedosphaerales *Pedosphaeraceae* NA | 0 | 1 | 0.800216687 | 0.011 | 0.017028249 | 0.000473437 | 0.000749098 |
| Bacteria Verrucomicrobiota Verrucomicrobiia Pedosphaerales Pedosphaeraceae SCGC AAA164-E04 | 0 | 1 | 0.856494584 | 0.006 | 0.009670588 | 0.000412647 | 0.001020179 |
| Bacteria Verrucomicrobiota Verrucomicrobiia Verrucomicrobiales *Rubritaleaceae Persicirhabdus* | 0 | 1 | 0.721671784 | 0.001 | 0.001802632 | 0.008982054 | 0.008797577 |

**Table S12. Network metric comparison for CC7-SSA01 and CC7-SSB01 lines under heat stress.** P-values were determined by generating 1000 random graphs using a Erdös-Rényi model, with the resulting graph’s metric values serving as our null distribution in two-tailed tests on the basis of z-scores. P-values represent metric comparisons between each line’s network.

| **Metric** | **P-value** |
| --- | --- |
| Density | 0.97938974 |
| Average path length | 0.03417697 |
| Transitivity | 0.11085568 |
| Maximum clique size | 0.81462447 |

**Table S13. Network metric comparison for CC7-SSA01 and CC7-SSB01 lines under heat stress as compared to random networks.** P-values were determined by generating 1000 random graphs using a Erdös-Rényi model, with the resulting graph’s metric values serving as our null distribution in two-tailed tests on the basis of z-scores. P-values represent metric comparisons between our observed and randomly generated networks.

| **Metric** | CC7-SSA01 | **P-value** | CC7-SSB01 | **P-value** |
| --- | --- | --- | --- | --- |
| Density | 0.7727273 | 9.911331e-01 | 0.4444444 | 9.786594e-01 |
| Average path length | 1.30303 | 1.360477e-01 | 1.803922 | 3.243276e-05 |
| Transitivity | 0.9285714 | 3.192082e-03 | 0.7211009 | 2.915525e-08 |
| Maximum clique size | 10 | 2.315908e-05 | 8 | 1.832239e-08 |

**
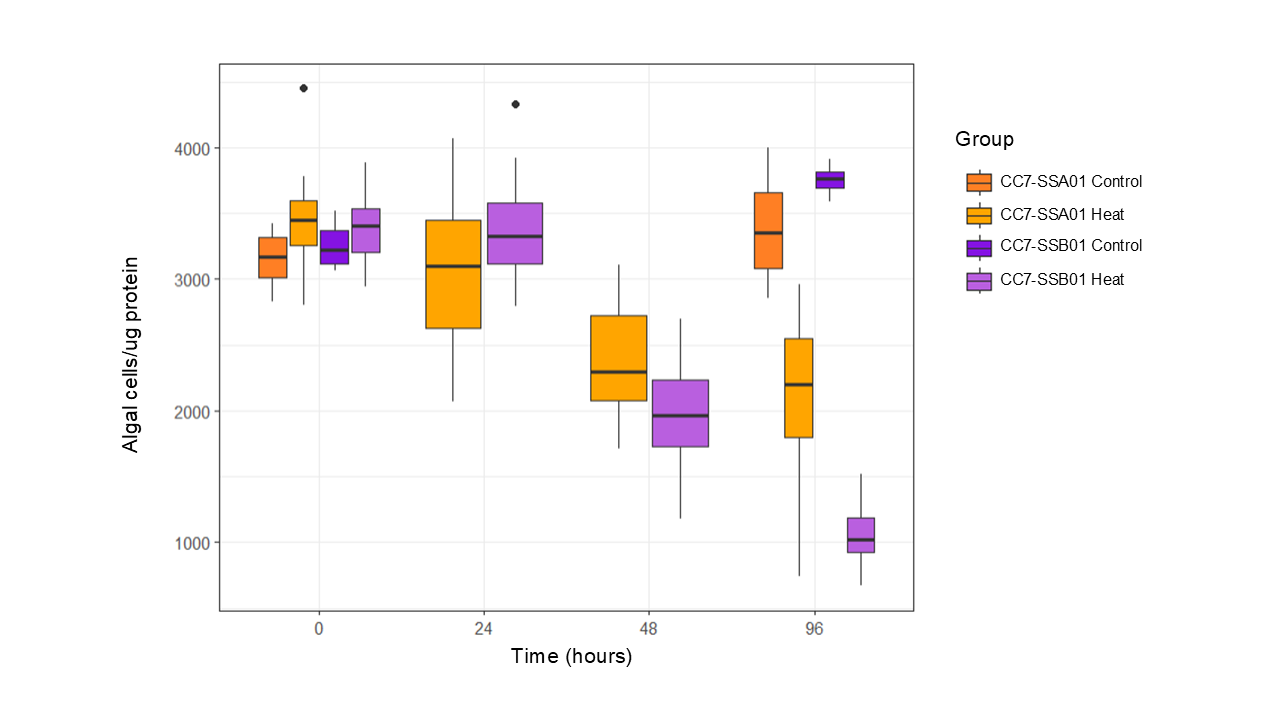
**

**Figure S1. Algal cell density over the course of our acute thermal stress assay.** Algal cell density is standardized per ug of host protein, serving as a proxy for bleaching severity; lower values indicate greater bleaching severity.
